# Understanding the development and implementation of national quality of care and patient safety strategic documents: a scoping review

**DOI:** 10.1186/s12913-025-13563-2

**Published:** 2025-11-27

**Authors:** Maria Mafalda Fernandes, Sofia Guerra-Paiva, Ana Raquel Soares, Rohanne Spiteri, Christos Triantafyllou, Konstantina Vasileiou, Marie Stridborg, Valter Fonseca, Joao Breda, Paulo Sousa

**Affiliations:** 1 Public Health Research Centre, Comprehensive Health Research Center, CHRC, NOVA University of Lisbon, Lisbon, Portugal; 2 WHO Athens Quality of Care and Patient Safety Office, World Health Organization Regional Office for Europe, Athens, Greece; 3 NOVA University of Lisbon, Public Health Research Centre, Comprehensive Health Research Center, CHRC, Lisbon, Portugal

**Keywords:** Quality of care, Patient safety, National policy documents, National health strategies, Scoping review

## Abstract

**Background:**

Several strategic documents aimed at integrating quality of care and patient safety into health systems have been developed, both as guiding documents produced by renowned international organisations and as national documents with a political purpose. However, the development, implementation and monitoring/evaluation of quality of care and patient safety strategic/policy documents are often delicate and challenging processes.

**Objectives:**

In order to support policymakers and decision makers who plan to develop or improve their national quality of care and patient safety strategic/policy documents, this study seeks to understand how the processes of developing, implementing, and monitoring/evaluating this type of policy have been carried out and how they should be conducted.

**Methods:**

A scoping review was conducted by searching three major databases for relevant articles and by examining official strategic documents published on the websites of national and governmental authorities. The methodology followed the stages approach proposed by Arksey and O’Malley and was guided by the Preferred Reporting Items for Systematic Reviews and Meta-Analysis Extension for Scoping Reviews.

**Results:**

Key phases and tasks across each stage of the development, implementation and monitoring/evaluation of strategic/policy documents relating to quality of care and patient safety were identified. The identification, consultation and involvement of various stakeholders emerged as cross-cutting factors across all three processes and were regarded as highly important.

**Conclusion:**

This study maps key tasks and phases relevant to the effective development, implementation, and monitoring/evaluation of strategic health policy documents. By outlining a structured approach aligned with international best practices, it offers actionable insights that may support policymakers in designing sustainable and high-impact policies. These findings contribute to strengthening planning for quality of care and patient safety, with potential implications for the delivery of safe, high-quality healthcare services and the development of resilient, responsive health systems.

**Registration number of the study:**

10.17605/OSF.IO/K4HXD.

**Supplementary Information:**

The online version contains supplementary material available at 10.1186/s12913-025-13563-2.

## Background

According to the World Health Organization (WHO) and the Institute of Medicine, the quality of health services should be effective, safe, people-centred, timely, efficient and equitable [1, 2]. However, patient harm resulting from unsafe healthcare is one of the leading causes of death and disability worldwide, and is therefore considered a significant and growing global public health challenge [3].

It is imperative to emphasise that the provision of unsafe healthcare is a problem throughout healthcare systems, that a large proportion of failures in the provision of healthcare are preventable, and that the frequency of preventable harm remains very high worldwide [3–5]. Additionally, the harm caused to patients has highly significant clinical, economic and social impacts [3, 5, 6].

Given this reality, quality of care and patient safety have become central topics on many countries’ agendas in recent decades. Patient safety is internationally recognised as a fundamental dimension of quality in healthcare and has undergone substantial development in recent decades [7]. It should also be seen as a human right and a fundamental principle of healthcare [4]. Recognising quality of care and patient safety as a priority in health sector policies is fundamental to achieving universal access to healthcare, which is not limited to guaranteeing access to healthcare but rather ensuring access to safe and high-quality healthcare [3–5].

To this end and following global advocacy in the realm of quality of care and patient safety, countries and international organisations have given particular attention to this topic. The WHO has led the agenda in this area through several initiatives over recent years, notably with the development of two significant documents that are used as strategic guides for countries to improve quality and safety within the healthcare sector: the *Handbook for national quality policy and strategy* (HNQPS) in 2018 and the *Global patient safety action plan 2021–2030* (GPSAP) in 2021 [3, 8]. The former aims to support countries in the process of developing and implementing national health quality policies and strategies, whereas the latter seeks to enhance patient safety globally by addressing preventable error [3, 8]. The GPSAP sets ambitious targets to reduce patient risks and outlines seven key strategic objectives [3].

Additionally, to ensure everyone receives the right care, including preventive and health promotion services, at the right time, in the right place, from the right person, without experiencing financial hardship, the WHO adopted the *framework for resilient and sustainable health systems for the WHO European Region 2025–2030* [9]. High-performing health systems, accessible to all people throughout the life course and responsive to a rapidly changing world, represent the realisation of this vision [9].

Several countries have already developed official strategic documents focused on improving quality of care and patient safety in their healthcare systems, which reinforces the importance that strategic planning for the evaluation and continuous improvement of quality of care and patient safety has had, both nationally and internationally [5].

This study aims to present the useful insights and considerations regarding the development, implementation and monitoring/evaluation of strategic/policy documents, emerging from the analysis of scientific literature and official policy documents from countries recognised for their contributions to advancing quality of care and patient safety.

This research can be of great value to countries planning to develop or improve their quality of care and patient safety policies, by providing insights that can support policymakers and decision-makers in better defining and implementing interventions in this field.

### Study rationale

The development, implementation and monitoring/evaluation of quality of care and patient safety strategic/policy documents (strategies, plans and programmes) are often delicate and challenging processes. There are several identified barriers that can compromise the decision making at different levels. In the end of 80s, Institute of Medicine had identified some of the core barriers, such as lack of data (monitoring) and knowledge to support the decisions, poor interaction and communication among technical and political aspects of decision and organizational fragmentation [10]. More recently, the identification of stakeholder resistance, scarce resources, lack of leadership support and adequate training are also identified as limitations to implement quality of care and patient safety policies/programmes and sustain them over time [11]. This type of challenges influence the effectiveness of policy and programmes implementation and sustainability.

Therefore, during the initial stages of these processes, the insights gathered from the scientific evidence and from other countries’ experiences can add considerable value and contribute to a more robust and smoother process. Given this, the present scoping review aimed to understand how the processes of developing, implementing, and monitoring and evaluation strategic/policy documents related to quality of care and patient safety have been and are expected to be conducted.

## Methodology

To achieve the aim of this study, a scoping review was conducted, focusing on the identification and mapping of the characteristics of strategic/policy documents (strategies, plans, and programmes) on quality of care and patient safety. These characteristics served as the basis for examining the processes of their development, implementation and monitoring/evaluation.

The methodology applied followed the approach proposed by Arksey and O’Malley [12] and was guided by the Preferred Reporting Items for Systematic Reviews and Meta-Analysis Extension for Scoping Reviews [13] to ensure the transparency of the results obtained.

### Stage 1: Identifying the research question(s)

To answer the main objective of this research, the following main question was defined:

- What are the main characteristics of the strategic/policy documents on quality of care and patient safety published in WHO European Region Member States, Australia, Canada and the United States of America?

To answer the main question, the following secondary questions were asked:

a) Who are the main identified stakeholders for the development of these strategies/plans/programmes?

b) What is the target population and settings of these strategies/plans/programmes?

c) What are the main priorities and objectives of these strategies/plans/programmes?

d) What are the main identified barriers to and facilitators of strategies/plans/programmes implementation?

e) What is the monitoring/evaluation process used to evaluate the achievement of the proposed objectives and actions?

### Stage 2: Search strategy

Three relevant databases (PubMed, Scopus and Web of Science) were searched with the necessary adjustments for each of the platforms (Table 1). The applied search strategy was developed with the support of a qualified research librarian. Last update of search strategy was made on 18th August 2025.

**Table 1:** Search strategies

Database	Last date of retrieval	Strategy
*Pubmed*	13/05/2024 Last update 18/08/2025	((“Quality of Health Care“[All Fields] OR “Patient Safety“[All Fields]) AND (“policies“[All Fields] OR “program“[All Fields] OR “strategies“[All Fields] OR “plan“[All Fields]) AND (“Federal Government“[All Fields] OR “national“[All Fields] OR “country“[All Fields]))
*Scopus*	13/05/2024 Last update 18/08/2025	TITLE-ABS-KEY ((“Quality of Health Care” OR “Patient Safety”) AND (“policies” OR “program” OR “strategies” OR “plan”) AND (“Federal Government” OR “national” OR “country”))
*Web of Science*	13/05/2024 Last update 18/08/2025	(“Quality of Health Care” OR “Patient Safety”) AND (“policies” OR “program” OR “strategies” OR “plan”) AND (“Federal Government” OR “national” OR “country”) (Topic) and Open Access and Article (Document Types)

To complement the database search, strategic/policy official documents on quality of health and patient safety were searched on the official websites of national and governmental authorities. Additionally, other relevant websites in the field, such as the Organisation for Economic Co-operation and Development website, the European Observatory for Health Systems website, the WHO website and the Global Knowledge Sharing Platform for Patient Safety, were searched.

### Stage 3: Study selection

Open access/full-text documents published between 2014 and 2024 were included. No language restrictions were applied to the inclusion of official policy documents, as the majority are written in their native languages. The included documents not written in English were translated into English via the artificial intelligence tool DeePL [14]. For the retrieval of scientific articles, a language restriction was applied, considering only articles published in English.

National-level documents were prioritised, as they were considered to provide the most comprehensive reflection of countries’ strategic approaches to quality of care and patient safety. However, in cases where such documents were not available, and/or where regional or local documents were judged to offer particularly relevant insights, these were also incorporated into the analysis. This approach allowed for the inclusion of a broader range of experiences, while maintain a primary focus on nationally endorsed strategic/policy documents.

Scientific articles and official strategic/policy documents that did not include clear information about the design/development, implementation or monitoring/evaluation process of the strategies/plans/programmes related to quality of care and patient safety were excluded. Additionally, those that targeted specific health conditions and that were not available online were also excluded.

In this study, we consider both processes of evaluation (periodic assessment to assess the effectiveness of interventions or strategies) and monitoring (ongoing data collection to track performance in a timely manner), since both are useful for assessing strategies/plans/programmes.

The articles/ official documents were independently and manually screened by two independent authors between June 2024 and September 2024. In the case of disagreement on article/document inclusion, a third author was involved to evaluate the paper independently and contribute to making a final decision. The screening, selection and data extraction were carried out by qualified members of the study team with prior experience in conducting literature reviews and in the fields of quality of care and patient safety.

### Stage 4: Charting the data

To collect the data, a framework was created to guide the extraction of information from grey literature and scientific articles, aiming to gather relevant insights and recommendations that could contribute to the design, implementation and monitoring of future national strategic/policy documents for quality of care and patient safety (Table 2).

This framework was constructed to guide the collection of data on the basis of the assumption that not all the domains would be completed in most of the collected documents/articles.

**Table 2:** Framework for data collection

Topics
Main Information (Document name, Authorship, Year of publication, Type of document, Country, Level [national, regional or local], Period of application, Target population, Target setting)
Development process (Stakeholders involved, Consultation process, Priorities, Objectives)
Implementation process (Actions, Barriers, Facilitators)
Monitoring process (Evaluation moments, Evaluators, Indicators)

### Stage 5: Collating, summarising and reporting the results

The framework was qualitatively analysed to describe the main characteristics of the included official strategic documents and scientific articles. Among the different dimensions, the target population, the development process (stakeholders involved, defined priorities and objectives), and the implementation and monitoring processes were analysed. The framework used for data collection was previously tested by the research team in a preliminary phase of data collection and adjusted to answer the needs of the study. For preliminary phase we have tested the search strategy in at least two databases as recommended by the Joanna Briggs Institute. We have tested the search strategy in Pubmed and Web of Science. Two independent researchers applied the search strategy and made the necessary adjustments. A third reviewer was involved in case of disagreements.

Additional file 1 presents the Preferred Reporting Items for Systematic reviews and Meta-Analyses extension for Scoping Reviews (PRISMA-ScR) Checklist.

## Results

**Fig. 1:**
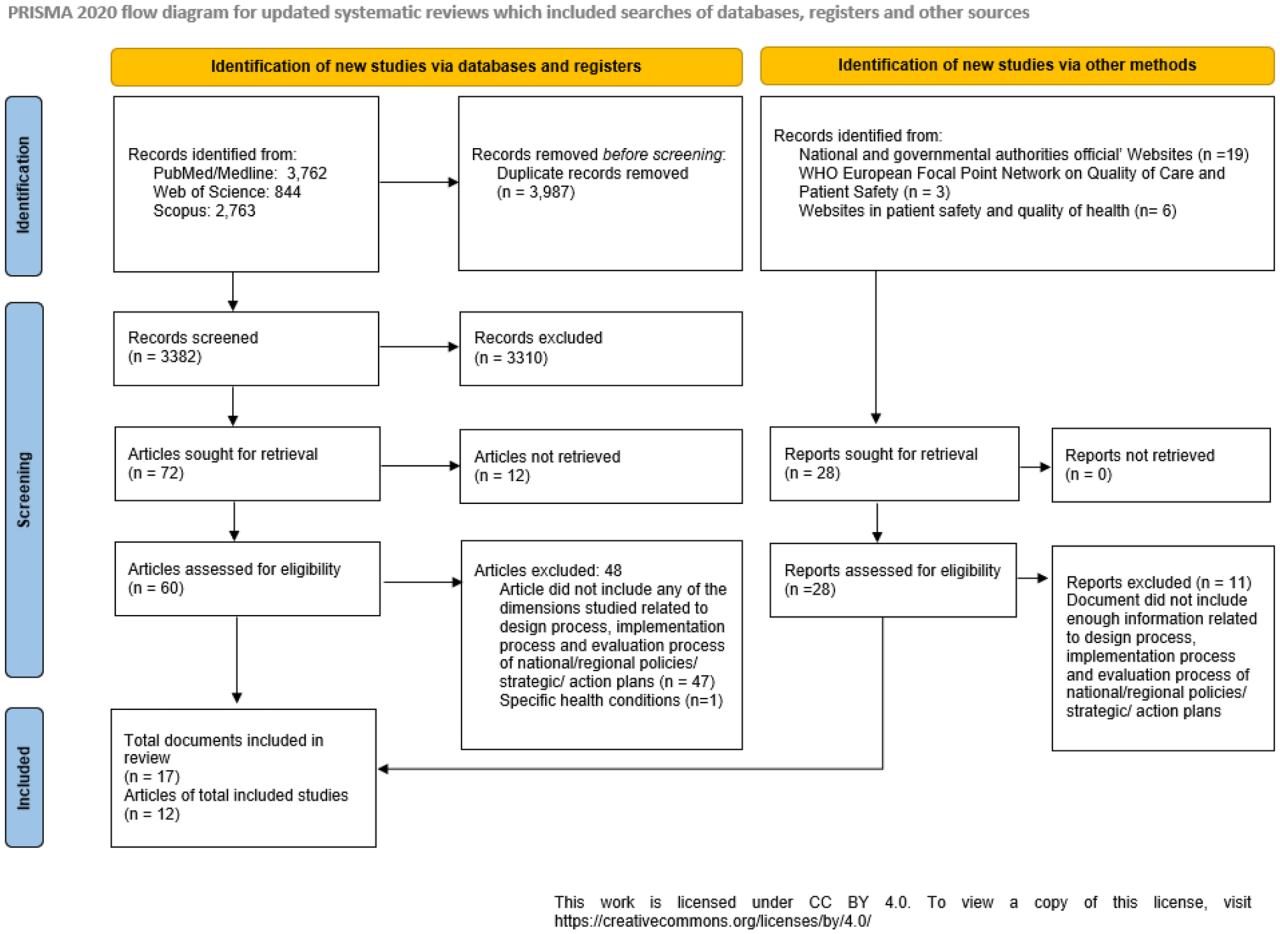
PRISMA flow diagram

A total of 7369 scientific articles were retrieved from the search strategy applied in PubMed, Scopus and Web of Science. Of these, 3987 articles were duplicated records and were consequently excluded. A total of 3382 articles were analysed for title and abstract by three independent researchers, and 72 articles were considered for full-text screening. By applying the inclusion and exclusion criteria, a total of 12 articles were selected for this study (Fig. 1).

In parallel, a total of 17 official strategic/policy documents (from 13 different countries) were analysed. All of them were produced by national organisations and/or ministries, and 13 were produced in the last five years. With respect to the application period, six documents lacked a defined period, four specified a five-year application period, and the others varied between two, four and eight years (Table 3).

Among the retrieved scientific articles and official documents, diverse strategies, plans and programmes were identified. Documents focused solely on quality of care or patient safety were identified, as were those focused on both topics (Tables 3 and 4).

**Table 3:** Main information of the 17 analysed official policy documents

Document (year of publication)	Authorship	Type of document	Country	Level	Period of application	Target population(s)	Target setting(s)
Patient safety strategy 2.0 (2018)	Federal Ministry of Labor, Social Affairs, Health and Consumer Protection	Strategy	Austria	National	Long-term validity. The Patient Safety Strategy 2.0 will be updated as necessary.	Policy/decision makers; state health funds; hospital providers; hospitals and all extramural health facilities in Austria; employers and people who are self-employed in a legally recognized health profession; citizens and patients in Austria	Healthcare system
Quality strategy for the Austrian healthcare system Version 2.1 (2022)	Federal Ministry of Labor, Social Affairs, Health and Consumer Protection	Strategy	Austria	National	2023. Constantly updating the strategy.	The strategy is aimed at the various groups in the healthcare system, i.e. decision-makers, financiers and healthcare service providers	Healthcare system
The Client and Patient Safety Strategy and Implementation Plan 2022–2026 (2022)	Ministry of social affairs and health	Strategy	Finland	National	4 years (2022–2026)	The strategy was prepared for use by healthcare and social welfare professionals, parties managing and supervising operations, political decision-makers, as well as clients, patients and their close ones. The strategy can be used by both public and private parties involved in healthcare and social welfare services.	Healthcare and social welfare services
National Patient Safety Programme (2013)	Ministry of Labor, Health and Solidarity	Programme	France	National	4 years (2013–2017)	The programme should enable all professionals, patients and users to identify the challenges of healthcare safety, the objectives to be pursued to guarantee safety and the means to improve, individually and collectively. Each player, whatever their role, is a link in the safety chain required to design and deliver care.	Healthcare system
1st roadmap 2023–2025 “Improving patient safety and residents”. A continuation of the national patient safety program 2013–2017 (2023)	Ministry of Labor, Health and Solidarity - Organization of the general direction of healthcare provision	Roadmap - action plan	France	National	2 years (2023–2025)	Ministry	Healthcare system
Patient Safety Strategy 2019–2024 (2019)	Health Service Executive (HSE)	Strategy	Ireland	National	5 years (2019–2024)	Patients, staff and other bodies such as the Department of Health, the Health Information and Quality Authority, the Mental Health Commission, the State Claims Agency and the Professional Bodies.	Health and social care services
National Plan for Patient Safety 2021–2026 (2021)	Directorate-General for Health	National Plan	Portugal	National	5 years (2021–2026)	Healthcare workers; managers; administrators	Healthcare facilities and institutions (public and private settings)
National Strategy for Health Quality 2015–2020 (2020)	Ministry of Health	Strategy	Portugal	National	5 years (2015–2020)	Patients and healthcare workers	Healthcare system
Patient Safety Strategy (2010)	Agency for accreditation of healthcare institutions in Serbia (AZUS)	Strategy	Serbia	National	Not defined	Accredited facilities in Serbia	Accredited healthcare facilities in Serbia
National Strategy for Patient Safety in Healthcare (2023–2031) (2023)	Ministry of Health	Strategy	Slovenia	National	8 years (2023–2031)	Healthcare professionals; patients and their families; researchers; payers; planners; educators	Healthcare facilities at all levels of care
Patient Safety Strategy for the National Health System 2015–2020 (2015)	Ministry of Health, Social Services and Equality	Strategy	Spain	National	5 years (2015–2020)	Patients within NHS; clinical professionals and management; healthcare organizations and providers; academic organizations and agents involved in enhancing patient safety in Spain.	All levels in all settings in which care is provided in the National Health System.
National Action Plan for Increased Patient Safety in Swedish Health Care 2020–2024: Act for safer healthcare (2020)	National Board of Health and Welfare	Action Plan	Sweden	National	4 years (2020–2024)	Principals’ decisionmakers. The Action Plan is designed to be used by municipalities and regions. Patients, healthcare workers, authorities, national organisations, representatives of higher education institutions, experts and politicians are also targeted.	All levels in all settings in which care is provided
The NHS Patient Safey Strategy (2019)	NHS England and NHS Improvement	Strategy	United Kingdom	National	5 to 10 years - updated regularly last on 2021	Healthcare workers; patients and families; managers	National Healthcare Services institutions
National Safety and Quality Health Service Standards − 2nd edition (2021)	Australian Commission on Safety and Quality in Health Care	National standards	Australia	National	Not defined	Leaders of a health service organisation and workforce	Healthcare service organizations
Improving safety and quality in health care - A strategic plan for action in WA 2024–2026 (2023)	Government of Western Australia - Department of health	Strategic plan	Australia	Regional	2 years (2024–2026)	Healthcare organization and workforce; patients	Healthcare settings
The Canadian Quality and Patient Safety Framework for Health Services (2020)	Health Standards Organization and Canadian Patient Safety Institute	Program/ Framework	Canada	National	Not defined	All people across Canada	All Canadian health systems
Safer Together: A National Action Plan to Advance Patient Safety (2020)	Institute for Healthcare Improvement - National Steering Committee for Patient Safety	Action Plan	United States of America	National	Not defined	Healthcare leaders; workforce; patients	The entire healthcare continuum

**Table 4:** Main information on the 12 analysed scientific articles

Article	Authors	Year of publication	Type of initiative addressed	Country	Level (National, Regional or Local)
National patient safety consortium: learning from large-scale collaboration	Sandi Kossey, Chris Power, Leslee Thomson, Kathleen Morris, Shelagh Maloney, Lee Fairclough, Deborah Prowse and Hina Laeeque	2020	Patient Safety Action Plan	Canada	National
A study of the implementation of patient safety policies in the NHS in England since 2000: what can we learn?	David Phillip Wood, Catherine A. Robinson, Rajan Nathan, Rebecca McPhillips	2022	Patient safety policy	United Kingdom (England)	National
The Better Care Plan: A blueprint for improving America’s healthcare system	Stephen M. Shortell, John T. Toussaint, George C. Halvorson, Jon M. Kingsdale, Richard M. Scheffler, Allyson Y. Schwartz, Peter A. Wadsworth, Gail Wilensky	2023	Quality Plan	United States of America	National
From accreditation to quality improvement-The Danish National Quality Programme.	Christian Uggerby, Solvejg Kristensen, Julie Mackenhauer, Søren Valgreen nudsen, Paul Bartels, Søren Paaske Johnsen, and Jan Mainz	2021	Quality Programme	Denmark	National
A Quality Strategy to Advance the Triple Aim in California’s Medicaid Program	Desiree R. Backman, Neal D. Kohatsu, Orion T. Stewart, Rachel L. Barrington, and Kenneth W. Kizer	2020	Quality Strategy	United States of America	Regional
Understanding the factors influencing implementation of a new national patient safety policy in England: Lessons from ‘learning from deaths’.	Lalani M, Morgan S, Basu A, Hogan H	2023	Patient Safety Policy	United Kingdom (England)	Local
Quality improvement lessons learned from National Implementation of the “Patient Safety Events in Community Care: Reporting, Investigation, and Improvement Guidebook”.	Jennifer L. Sullivan, Marlena H. Shin, Jeffrey Chan, Michael Shwartz, Edward J. Miech, Ann M. Borzecki, Edward Yackel, Sachin Yende, Amy K. Rosen	2024	Patient Safety Strategy	United States of America	Local
The Danish health care quality programme: Creating change through the use of quality improvement collaboratives.	Carstensen K, Kjeldsen AM, Lou S, Nielsen CP	2022	Quality Programme	Denmark	Local
Reporting and use of the OECD Health Care Quality Indicators at national and regional level in 15 countries	Alexandru M. Rotar, Michael J. Van Den Berg, Dionne S. Kringos, Niek S. Klazinga	2016	Use of OECD quality indicators	15 Countries - OECD member states	National
Quality improvement and accountability in the Danish health care system	Jan Mainz, Solvejg Kristensen, Paul Bartels	2015	Quality Strategies	Denmark	National
Effects of the Italian Law on Patient Safety and Health Professional Responsibilities Five Years after Its Approval by the Italian Parliament	Giuseppe Candido, Fidelia Cascini, Peter Lachman, Micaela La Regina, Chiara Parretti, Valentina Valentini, Riccardo Tartaglia	2023	Patient Safety Policy	Italy	National
Analysing ‘big picture’ policy reform mechanisms: the Australian health service safety and quality accreditation scheme	David Greenfield, Reece Hinchcliff, Margaret Banks, Virginia Mumford, Anne Hogden, Deborah Debono, Marjorie Pawsey, Johanna Westbrook, Jeffrey Braithwaite	2014	Quality Policy	Australia	National

Various stakeholders were identified in the design process of the initiatives analysed. Common stakeholders mentioned in the scientific articles, included government bodies, healthcare providers, regulators, professional organisations and patient representatives (see Additional file 2). The Canadian initiative [15] involved the creation of a national consortium with 50 organisations from various sectors and various parts of Canada, whereas the Californian quality strategy [16] involved more than 5,000 stakeholders, including internal employees of the department leading the development of the initiative, external experts (mostly from academia) and a statewide working group. In an Australian initiative, the consultation process lasted five years, during which regulators, accreditation agencies, insurers and consumers were consulted [17].

For this design/development process, a consultation phase was also noted in most of the included official documents (14/17 documents). Several stakeholders contributed to the development of these initiatives across different healthcare systems and regions, with 13 initiatives involving national and regional stakeholders in this process. Among the different partners, patient safety advisory boards, representatives of national organisations and regional administrations, national and regional bodies, and partnerships with patients were the most common collaborations. The information above can be found in the Additional file 3.

All the initiatives analysed (within the scientific articles and official documents) are focused on responding to patients’ and healthcare workers’ needs. However, it was possible to observe in the official documents that the majority (14/17 documents) encompass other groups, such as families, policymakers, hospital/health facility administrators and educators (see Table 4).

With respect to the main priorities/objectives defined for the initiatives described in the analysed scientific articles, patient safety emerged as the most frequently reported aspect (6/12 articles). Other recurrent themes included continuous quality improvement, transparency of results, equity and access, and efficiency and sustainability of the health system (all present in 3/12 articles). Less frequently mentioned were patient involvement and integration and coordination of care (both present in 2/12 articles) (see Additional file 2).

When analysing the official documents, although the strategies/plans/programmes vary across different countries, several common priority areas have also been identified, with patient safety emerging as the most consistently emphasised priority (13/17 documents). Other aspects frequently highlighted included patient and family involvement (8/17 documents), enhancing the safety culture (6/17 documents), quality measurement and transparency (5/17 documents), and risk management and reporting systems (5/17 documents). Several additional dimensions were also recurrent, namely continuous learning systems, evidence-based practices, people-centered care, and training and capacity building (all appearing in 4/17 documents). Less frequently, but still present in multiple documents, were integration and coordination of care, equity and access, clinical governance and organisational development (each in 3/17 documents), and workforce safety and wellbeing (2/17 documents). Regarding main objectives, a focus on areas such as safety culture and patient engagement, followed by medication safety, healthcare associated infections and communication was noted (see Additional file 3 and Additional file 4).

Almost all of the analysed official documents identified and described measures/actions to achieve the established objectives (16/17 documents). Certain measures identified were designed to allow for adjustment to regional needs while others were specifically tailored for implementation at the regional or local level.

Actively engaging with different stakeholders and leadership involvement were frequently reported in the included scientific articles as facilitator factors for the implementation process (reported in 8 and 5 out of 12 articles, respectively). Clearly defining the purpose, roles, and responsibilities of stakeholders/collaborators is seen as essential for building a common understanding and facilitating communication, as it guides stakeholders throughout the implementation process. Some of the main recommendations included (1) identify and train “champions” to advocate the program; (2) Build partnerships and connect teams; (3) Obtain formal commitments; (4) Involve executive boards in the implementation process; and (5) establish an external multidisciplinary advisory committee [15, 18, 19]. Systems for reporting, monitoring and transparency as well as capacity building were also reported as facilitators (mentioned in 9 and 7 out of 12 articles, respectively). Countries such as Australia [17] and Denmark [20] particularly highlight the importance of assessing the compliance of institutions with quality improvements to guide the implementation process.

On the other hand, as barriers for the implementation process, lack of financial support, resistance to change, top-down approach, and difficulties in coordinating and following up procedures were reported as constraining factors (each reported in 1/12 articles) (see Additional file 5).

In the official documents, the identified facilitating and constraining factors seem to be in line with those reported in the scientific articles. Facilitating factors were identified in 10 out of 17 documents and aspects such as collaboration and coordination among stakeholders, workforce (healthcare workers and other professionals) competence and wellbeing, and leadership and administrative commitment were mentioned (reported in 3, 3 and 1 articles, respectively). Barriers for the implementation were only identified in 6 out of 17 documents, with the most prominent aspect being the lack of resources, both human and material (4/17 documents). Other aspects included cultural challenges, an overly macro-level focus, insufficient education and training, and the COVID-19 pandemic (each reported in 1/17 documents) (see Additional file 6).

Survey applications, followed by interviews and focus groups, were reported as tools to monitor/evaluate the effectiveness of the initiatives described in the scientific articles (reported in 3, 1 and 1 articles out of 12, respectively), as well as quantitative data collection, often linked to quality indicators (reported in 3/12 articles). This last process involved a panel of indicators that was used at the national, regional and hospital levels [20]. Reference to the creation of a specific multidisciplinary team responsible for the evaluation process was made in an article, which focused on evaluating results and the implementation process [15]. The provision of access to the evaluation results was largely appointed by the scientific articles as an important feature of the monitoring process (see Additional file 7).

When analysing the official documents, most of them (13/17 documents) were not clear regarding the evaluation process, specifically on how, when and by whom the evaluation should be conducted. Although most documents do not specify evaluation indicators, those that do so (5/17 documents) align them with the specific objectives of the strategies. Only two out of 17 official documents provided information regarding when the monitoring/evaluation process should take place; however, they simply stated that evaluations should be performed regularly, without specifying exact intervals for this purpose. Despite the absence of a clearly defined evaluator in most documents, eight out of 17 have identified institutions, designated commissions or the document’s authors themselves as responsible for the evaluation. The above information can be consulted in the Additional file 8.

## Discussion

The present scoping review included 29 records focused on quality of care and patient safety strategies, plans, and programmes. These are typically supported by operational plans assigning clear responsibilities across health system levels and setting priorities around patient safety, workforce wellbeing, people-centred care, and system integration. Stakeholder consultation emerged as a common feature of these documents. Although the analysed documents appear to align with international recommendations, they also note gaps in the monitoring and evaluation processes, particularly in defining responsibilities, methods, metrics and indicators. Strengthening these aspects is essential to ensuring the long-term effectiveness and sustainability of health quality policies.

Following the analysis conducted, the findings suggest that the development of strategies/plans/programmes in quality of care and patient safety often involves a consultation process with various stakeholders. Among the different partners identified for this purpose, government bodies (at the national and regional levels), representatives of national and independent organisations, healthcare providers, and patients and their families were the most commonly involved in the initiatives analysed. This process reflects a collaborative approach aimed at incorporating diverse perspectives and ideas to develop comprehensive and evidence-based solutions. By involving a broad spectrum of stakeholders, these processes can seek to ensure that the resulting policies are practical, widely supported and centred on improving health systems and patient outcomes.

These findings appear to be consistent with established scientific evidence on the importance of stakeholder engagement in health and healthcare guideline development. According to the international Multi-Stakeholder Engagement (MUSE) Consortium, involving a diverse range of stakeholders throughout the entire process of guideline development not only enhances the relevance and transparency of the guidelines but also improves their acceptability and feasibility [21]. This involvement is seen as crucial for identifying, selecting and addressing priority health issues and has been shown to increase uptake and adherence to health/healthcare recommendations, ultimately improving health outcomes [21]. Additionally, the WHO states in the HNQPS that a quality strategy must involve a structured, data-driven and multistakeholder process [8]. For a quality strategy to be successful, meaningful collaboration and involvement of stakeholders is crucial not only in the design phase but also in the implementation and evaluation phases. The MUSE acknowledges that successful guideline development and implementation needs the engagement of multiple stakeholders and shared solutions [21].

Regarding stakeholder involvement, the literature emphasizes patient, public and community engagement [21]. The stakeholders identified in this study’s analysis appear to be in line with this and with the stakeholder groups identified in the HNQPS as those generally depicted in the development of quality strategies [8]: (i) government (ministries [health and related non-health, such as finance], quasi-governmental arms-length bodies and key elected officials); (ii) regulators and other external evaluators and standard-setting bodies; (iii) public and private insurance entities and authorities; (iv) professional societies; (v) providers (community, primary, secondary and tertiary care, traditional medicine providers, public and private sectors); (vi) civil society organisations, large faith-based organisations, patient groups and patients; (vii) nongovernmental organisations and community-based organisations; (viii) payers, funders and donors.

According to the analysis conducted, while the initiatives most often target the public health setting, the social sector may also be mentioned in the design of these strategic documents, particularly in countries where the ministries of health and social security fall under the same administration. Furthermore, although the target population of these initiatives consists mainly of patients and healthcare professionals, achieving the defined objectives/goals may require the involvement of a broader range of stakeholders. As identified in the initiatives analysed, other groups - such as families, hospital and healthcare facility administrators, and policymakers (as mentioned above) - also play prominent roles, with actions directed towards them or responsibilities assigned to them.

Furthermore, the documents seem to outline the main priorities/goals of the initiatives clearly and prominently. This is broadly in line with the WHO recommendations for the development of strategies, since assessing a country’s current national health goals and priorities, identifying gaps and suggesting new objectives for the national health quality strategy are considered vital steps for the success of the policy [8]. The definition of priorities is seen as essential, since a strategy on quality of care seeks to ensure that the health system meets international and national objectives, and to guarantee alignment with national priorities, avoiding a fragmented approach [8].

In the different strategies/plans/programmes analysed, the main priorities tend to converge, with a common focus on the following topics: patient safety, workforce safety and wellbeing, continuous quality improvement, continuous learning systems, quality measurement and transparency, integration and coordination of care, people-centred care, and patient and family involvement. Interestingly, these priorities may reflect one of the types of priorities that the WHO highlights as being relevant in countries’ strategies, namely those focused on improvement in specific dimensions of quality, such as effectiveness, safety or people-centredness [8].

It is also worth noting that based on the identified main priorities and goals, the seven strategic objectives of the WHO Global patient safety action plan 2021–2030 [3] appear to be reflected in the initiatives analysed. Among these, the strategic objectives most reflected are: High-reliability health systems; Safety of clinical processes; Patient and family engagement, and Health worker education, skills and safety. Moreover, a connection can be observed with the six International Patient Safety Goals defined by the Joint Commission International [22], as objectives related to safe medication and hospital-acquired infections are also covered in the records analysed.

There seems to be a general need for strategic documents of this kind to include/define concrete actions to achieve the objectives set. This is supported by the fact that all the initiatives analysed, with the exception of one [23], outlined specific actions to be undertaken. In addition to actions designed for implementation at the national level, the analysis conducted identified the description of actions intended for the regional and local levels. Furthermore, some initiatives went even further by assigning the respective stakeholder(s) to be involved in the implementation of each action.

This definition of concrete actions, as well as the assignment of actions and responsibilities to specific stakeholders, aligns with what is referred to as an ‘operational plan’ in the HNQPS [8]. According to this document, a national quality strategy should be supplemented with an operational plan that defines clear milestones and tasks that must be undertaken, clarifies roles and responsibilities, sets clear timelines, and addresses financial and resource considerations [8]. The description of actions to be implemented at the national/regional/local level is also mentioned for this operational plan. Given that collaboration and commitment across all levels of the health system are essential for developing the operational plan (to ensure that the defined objectives are met and, consequently, the success of the health strategy), specific actions can be allocated across the national, regional, district, community, and facility levels [8]. Thus, an operational plan is seen as crucial in facilitating the strategy’s dissemination and implementation [8]. On the basis of all these findings, it appears that there is a definition of an operational plan within the analysed documents, suggesting that countries are making an effort to follow the recommendations for developing a successful health policy (specifically, in quality of care and patient safety).

Scientific evidence indicates that monitoring and evaluation enable healthcare managers/leaders and policymakers to enhance the effectiveness and positive impact of interventions by overseeing the project and identifying areas for improvement. According with the United Nations Development Group, there should be regular and systemic assessment of performance (monitoring), which is highly important for tracking strategies/plans/programmes, compared them with planned results as well as supporting decision making in immediate manner. Moreover, this should be complemented with evaluation, which refers to an impartial and systematic assessment to assess the overall impact and determines its efficiency and effectiveness [24]. Both monitoring and evaluation processes allow the assurance of the project’s sustainability and the estimation of the impact that can be attributed to a particular initiative/intervention, allowing for evidence-based decision-making and justification of resource allocation [25–28]. Furthermore, the WHO recognises that a quality strategy should include a plan for quality monitoring, feedback and overall evaluation of what progress is being made against the national goals [8].

However, as previously mentioned, the analysis conducted did not allow for a concrete conclusion regarding who has been, or should be, responsible for the monitoring and evaluation process, or how or when it has taken place or should take place, given that limited information was identified on these points in the documents analysed and that no consensus on these aspects was observed among the various initiatives. In general, it was only possible to identify diverse evaluation methods, both qualitative (e.g., interviews and focus groups) and quantitative (e.g., evaluation of quality indicators and data collection), multidisciplinary teams and designated commissions responsible for conducting the process, as well as recommendations for its periodic and regular execution and for the dissemination and transparency of its results. Nevertheless, it is noteworthy that the information gathered appears to align with what is presented in the literature focusing on the monitoring and evaluation of health interventions [8, 26, 29, 30].

According to relevant published sources in this domain, there seems to be a consensus that various stakeholders should play a pivotal role in this monitoring/evaluation process through significant collaboration and involvement [8, 29, 30]. This process should be led by the country’s health authorities and conducted by a team of qualified evaluators, who should work collaboratively with diverse stakeholders, with the view and opinion of civil society organisations/groups being considered fundamental [8, 29, 30]. Consequently, it will include individuals with a broad range of perspectives derived from both lived and professional experiences, as well as those directly involved in or impacted by the initiative’s outcomes [29]. Additionally, robust metrics are considered essential for quality improvement initiatives and both quantitative and qualitative methods are commonly used such as quantitative dashboards, focus groups and interviews to collect data [31].

The WHO defined the patient safety core indicator survey to collect a group of metrics to be applied in the WHO member states [32]. The application of patient safety programmes at national level is one of the core indicators, which highlights the importance of understanding the different factors that should be considered in their development, implementation and evaluation/monitoring processes on national, regional and local levels. This will allow to decrease the gap between the planning, effective implementation and sustainability of the policies/programmes/plans.

Furthermore, the literature indicates that this monitoring/evaluation process comprises several phases, which include, among other activities, understanding the context of the initiative, defining and assessing evaluation indicators, consulting stakeholders and acting on findings and recommendations [29, 30]. The literature reinforces the involvement of all stakeholders throughout various phases of the monitoring and evaluation process to ensure its success and, consequently, the success of the initiative in question [8, 30]. The definition of suitable metrics/indicators and their application in a timely manner is considered crucial for conducting a rigorous monitoring and evaluation process [26].

Lastly, and regarding the “when”, the evidence suggests that monitoring and evaluation should be ongoing activities integrated into the planning, implementation and post-implementation phases of the interventions developed. This continuity and regular execution ensures that interventions remain aligned with their objectives and allow for the necessary adjustments to be made in a timely manner [29, 30].

While the information identified during the analysis of the scientific articles and official documents appears to be in accordance with the evidence regarding this process, the apparent lack of information on monitoring and evaluation steps in most of the documents may indicate a weakness in the official documents analysed.

When designing these strategic documents, it seems to be essential to consider the factors that may facilitate their implementation, sustainability and success, as well as those that could negatively impact or constrain them. This affirmation is based not only on the analysis performed but also on scientific evidence indicating the existence of multiple factors (contextual) that interfere with the implementation of health and healthcare interventions [33–36]. Furthermore, evidence on what a health strategy’s operational plan involves also indicates that it must address financial and resource considerations, as mentioned above, and incorporate mechanisms for monitoring and addressing potential challenges that may arise during the implementation phase [8].

The collection and dissemination of monitoring and evaluation data was identified through the analysis conducted as a facilitating factor. Literature focused on health programmes’ monitoring and evaluation supports this idea that systematic data collection, sharing, and transparency with stakeholders must be part of the monitoring/evaluation process, contributing positively to the success of the initiatives, as it is crucial for continuous adjustments, feedback, transparency and engagement [8, 29, 30].

In addition, the analysis conducted also highlighted factors such as leadership involvement/commitment, collaboration and coordination among all the stakeholders, capacity building/workforce competence and workforce wellbeing as facilitator factors that may be considered (by policymakers and other stakeholders) in the design and implementation of such strategic documents. The publication of the document “Global strategy on human resources for health: Workforce 2020” reinforces the importance of addressing the issue of the healthcare workforce to provide a quality healthcare service and achieve the objectives of the national strategies on quality of care and patient safety [37]. Furthermore, lack of financial, human, and material resources, resistance to change from certain stakeholders, as well as top-down approaches/macro-level focus are some of the limiting factors that, according to the analysis performed, may arise. It is advisable that policymakers and responsible teams formulate practical strategies to navigate these challenges, as the literature indicates [8].

### Limitations

A limitation of this study is the significant variation in the structure, alignment and terminology used between all the documents and scientific articles analysed. Notably, identifying the information that corresponded to the priority areas and the main objectives and differentiating between them was challenging due to the lack of clarity and standardization related with the methodologic structure of the documents. The same scenario was also present when differentiating between the main and the specific objectives. The terminology used to designate the actions defined and the way in which they were presented sometimes differed across the diverse documents and may have been confused with strategic objectives (in some cases). Although we have tried to overcome the heterogeneity among the documents, through a structured framework for data extraction, this variability can hinder the comparison and analysis of strategies, plans and programmes, which can affect the accuracy of the results of this study. Based on these limitations we strongly recommend the use of an international methodologic guideline to overcome the lack of clarity and stablish a consensus on the methodology and structure of the strategic/policy documents among different countries.

Additionally, the use of policy documents from WHO European Region Member States, Australia, Canada, and the United States of America – given these countries’ demonstrated experience in the fields of quality of care and patient safety – introduces limitations to the findings of our study, particularly with regard to their comparability with contexts from low-income countries. Future research should extend this analysis to include policy documents from low- and middle-income countries, in order to capture a broader diversity of health system contexts and to enhance the generalisability and applicability of the findings across different settings.

Although quality assessment of included studies is not a mandatory criterion in scoping review methodology, it is important to note that its absence can reduce transparency and potentially influence the conclusions of the review. In this study, due to the heterogeneity of the included studies and documents, we chose not to assess their quality, as this would have hindered reliable comparisons by requiring the use of different quality assessment tools.

## Conclusion

The successful development of quality of care and patient safety strategic/policy documents relies on a structured approach encompassing three main processes: design, implementation, and monitoring/evaluation. This study, through an analysis of official policy documents and scientific evidence, identifies key aspects of these processes, specifically for strategies, plans and programmes in quality of care and patient safety.

Regarding the design process, steps such as the identification of priorities, objectives, and actions should take place, for which the sharing of visions, opinions, and experiences from various stakeholders is essential. The scientific evidence focused on quality of care and patient safety, which encompasses various international recommendations, should also be considered during these steps. For each of these steps, there are multiple aspects to reflect on, which are highlighted in this study.

Accounting for the various positive and negative factors that may influence the implementation of a quality of care and patient safety strategic/policy document and formulating measures to capitalise on opportunities or address challenges, is an important element of the implementation process. This scoping review also highlights that the involvement of all stakeholders in a co-design approach is frequently highlighted as an important facilitator for this phase. Collaboration and coordination among stakeholders, wellbeing of healthcare professionals, financial limitations and resistance to change are among the positive and negative factors identified in this study, respectively. A co-design approach involving all stakeholders can contribute to overcoming barriers, promoting collaboration and alignment across healthcare systems.

The monitoring and evaluation process enables the assessment of whether the set actions and objectives are being achieved, allows for necessary adjustments and can, consequently, contribute to the initiative’s effectiveness and sustainability. The definition and use of evaluation indicators is crucial for this process. Additionally, the sharing and transparency of monitoring and evaluation results with all the stakeholders involved is often regarded as fundamental to supporting the implementation and assessment phases.

The findings of this work highlight that the consultation and involvement of diverse stakeholders is identified as an important and recurrent factor across the three processes inherent to the successful development of quality of care and patient safety strategic/policy documents.

This scoping review reflects an effort to make policies evidence-based and structured for implementation and provides insights for countries seeking to develop or enhance quality of care and patient safety strategic documents. By focusing on these core processes (and their phases) and prioritising stakeholder engagement, healthcare systems can implement more sustainable and high-impact strategies aligned with international best practices. These policy/strategic documents have the potential to contribute to safer, patient-centred, and more efficient, equitable, accessible, and effective care.

## Supplementary Information

Below is the link to the electronic supplementary material.

Supplementary Material 1: PRISMA-ScR_Checklist

Supplementary Material 2: Information on the development process mentioned in the 12 articles

Supplementary Material 3: Information on the development process of the 17 analysed documents

Supplementary Material 4: Information on the development process of the 17 analysed documents (main objectives)

Supplementary Material 5: Information on the implementation process mentioned in the 12 articles

Supplementary Material 6: Information on the implementation process of the 17 analysed documents

Supplementary Material 7: Information on the evaluation/monitoring process mentioned in the 12 articles

Supplementary Material 8: Information on the evaluation/monitoring process of the 17 analysed documents

## Data Availability

The datasets supporting the conclusions of this article are included within this article and its additional files.

## Abbreviations

GPSAP: Global patient safety action plan 2021–2030
HNQPS: Handbook for national quality policy and strategy
MUSE: Multi-Stakeholder Engagement Consortium
PRISMA: Preferred Reporting Items for Systematic reviews and Meta-Analyses
WHO: World Health Organization

## References

[1] World Health Organization. Quality of care [Internet]. Available from: https://www.who.int/health-topics/quality-of-care

[2] Institute of Medicine (US) Committee on quality of health care in America. Crossing the quality chasm: a new health system for the 21st Century [Internet]. Washington (DC): National Academies Press (US). 2001. Available from: http://www.ncbi.nlm.nih.gov/books/NBK222274/25057539

[3] World Health Organization. Global patient safety action plan 2021–2030: towards eliminating avoidable harm in health care [Internet]. World Health Organization. 2021. Available from: https://iris.who.int/handle/10665/343477

[4] World Health Organization. Regional office for the Eastern Mediterranean. Patient safety assessment manual: third edition, 3 ed. [Internet]. 2020. Available from: https://iris.who.int/handle/10665/363992

[5] Sousa P, Paiva SG, Lobão MJ, Van-Innis AL, Pereira C, Fonseca V. Contributions to the Portuguese National plan for patient safety 2021–2026: A robust methodology based on the Mixed-Method approach. Port J Public Health. 2022;39(3):175–92. 39469310 10.1159/000521722 PMC11320079

[6] World Health Organization. Patient safety [Internet]. Available from: https://www.who.int/news-room/fact-sheets/detail/patient-safety

[7] Sousa P, Van Innis A, Pereira LA, Lobão MJ, Fortuna Faria M, Guerra Paiva S. Relatório executivo: proposta de Plano Nacional Para a Segurança Dos Doentes 2021–2026. Lisboa: Escola Nacional de Saúde Pública. Universidade Nova de Lisboa. 2021.

[8] World Health Organization. Handbook for national quality policy and strategy: a practical approach for developing policy and strategy to improve quality of care [Internet]. World Health Organization. 2018. Available from: https://iris.who.int/handle/10665/272357

[9] Regional Committee for Europe 74th session. Seventy-fourth Regional Committee for Europe: Copenhagen, 29–31 October 2024: framework for resilient and sustainable health systems in the WHO European Region 2025–2030 [Internet]. World Health Organization. Regional Office for Europe. 2024. Available from: https://iris.who.int/handle/10665/378418

[10] The Future of Public Health [Internet], Washington DC. National Academies Press; 1988 [cited 2025 Aug 22]. Available from: http://www.nap.edu/catalog/1091

[11] Tappen RM, Wolf DG, Rahemi Z, Engstrom G, Rojido C, Shutes JM, et al. Barriers and facilitators to implementing a change initiative in long-term care using the INTERACT^®^ Quality Improvement Program. Health Care Manag [Internet]. 2017 Jul [cited 2025 Aug 22];36(3):219–30. Available from: https://journals.lww.com/00126450-201707000-00003 10.1097/HCM.0000000000000168 PMC5533173 28650872

[12] Arksey H, O’Malley L. Scoping studies: towards a methodological framework. Int J Soc Res Methodol. [Internet]. 2005;8(1):19–32. Available from: http://www.tandfonline.com/doi/abs/10.1080/1364557032000119616

[13] Tricco AC, Lillie E, Zarin W, O’Brien KK, Colquhoun H, Levac D, et al. PRISMA Extension for Scoping Reviews (PRISMA-ScR): checklist and explanation. Ann Intern Med [Internet]. 2018;169(7):467–73. Available from: 10.7326/M18-0850https://www.acpjournals.org 10.7326/M18-0850 30178033

[14] DeepL SE. DeepL Translator [Internet]. DeepL SE; Available from: https://www.deepl.com/translator

[15] Kossey S, Power C, Thomson L, Morris K, Maloney S, Fairclough L, et al. National patient safety consortium: learning from Large-Scale collaboration. Healthc Q. 2020;22(SP):10–26. 32049612 10.12927/hcq.2020.26050

[16] Backman DR, Kohatsu ND, Stewart OT, Barrington RL, Kizer KW. A quality strategy to advance the triple aim in california’s medicaid program. Am J Med Qual. 2020;35(3):213–21. 31272192 10.1177/1062860619860251

[17] Greenfield D, Hinchcliff R, Banks M, Mumford V, Hogden A, Debono D, et al. Analysing ‘big picture’ policy reform mechanisms: the Australian health service safety and quality accreditation scheme. Health Expect. 2015;18(6):3110–22. 25367049 10.1111/hex.12300 PMC5810648

[18] Wood DP, Robinson CA, Nathan R, McPhillips R. A study of the implementation of patient safety policies in the NHS in England since 2000: what can we learn? J Health Organ Manag. 2022;ahead-of-print(ahead-of-print) 10.1108/JHOM-02-2021-0073 35298879

[19] Lalani M, Morgan S, Basu A, Hogan H. Understanding the factors influencing implementation of a new National patient safety policy in england: lessons from ‘learning from deaths’. J Health Serv Res Policy. 2023;28(1):50–7. 35521697 10.1177/13558196221096921 PMC9850371

[20] Uggerby C, Kristensen S, Mackenhauer J, Knudsen SV, Bartels P, Johnsen SP, et al. From accreditation to quality improvement-The Danish National quality programme. Int J Qual Health Care. 2021;33(2):mzab071. 33861335 10.1093/intqhc/mzab071

[21] Petkovic J, Riddle A, Akl EA, Khabsa J, Lytvyn L, Atwere P, et al. Protocol for the development of guidance for stakeholder engagement in health and healthcare guideline development and implementation. Syst Rev. 2020;9(1):21. 32007104 10.1186/s13643-020-1272-5 PMC6995157

[22] Joint Commission International. International Patient Safety Goals [Internet]. Available from: https://www.jointcommissioninternational.orghttps://www.jointcommissioninternational.org/standards/international-patient-safety-goals/

[23] Patient Safety and Clinical Quality Directorate; Department of Health 3. Improving safety and quality in health care: a strategic plan for action in WA 2024–2026 [Internet]. 2024. Available from: https://www.health.wa.gov.au/Reports-and-publications/Improving-safety-and-quality-in-health-care

[24] United Nations Development Group. UNDAF Campanion Guidance: Monitoring and Evaluation. [Internet]. 2017. Available from: https://unsdg.un.org/sites/default/files/UNDG-UNDAF-Companion-Pieces-6-Monitoring-And-Evaluation.pdf

[25] Guerra-Paiva S, Lobão MJ, Simões DG, Fernandes J, Donato H, Carrillo I, et al. Key factors for effective implementation of healthcare workers support interventions after patient safety incidents in health organisations: a scoping review. BMJ Open. 2023;13(12):e078118. 38151271 10.1136/bmjopen-2023-078118 PMC10753749

[26] Pan American Health Organization. Health Indicators. conceptual and operational considerations. 2018; Available from: https://iris.paho.org/handle/10665.2/49056

[27] Clarke GM, Conti S, Wolters AT, Steventon A. Evaluating the impact of healthcare interventions using routine data. BMJ. 2019;365:l2239. 31221675 10.1136/bmj.l2239 PMC6584784

[28] Mainz J. Developing evidence-based clinical indicators: a state of the Art methods primer. Int J Qual Health Care. 2003;15(Suppl 1):i5–11. 14660518 10.1093/intqhc/mzg084

[29] Kidder DP, Fierro LA, Luna E, Salvaggio H, McWhorter A, Bowen SA, et al. CDC Program Evaluation Framework. 2024;73(6). 10.15585/mmwr.rr7306a1 PMC11444645 39316770

[30] International Health Partnership, World Health Organization. Monitoring, evaluation and review of national health strategies: a country-led platform for information and accountability [Internet]. Geneva: World Health Organization. 2011. 49 p. Available from: https://iris.who.int/handle/10665/85877

[31] Murphy DR, Savoy A, Satterly T, Sittig DF, Singh H. Dashboards for visual display of patient safety data: a systematic review. BMJ Health Care Inform [Internet]. 2021 Oct [cited 2025 Aug 22];28(1):e100437. Available from: 10.1136/bmjhci-2021-100437https://informatics.bmj.com/lookup/doi/10.1136/bmjhci-2021-100437 PMC8496385 34615664

[32] World Health Organization. The Global Health Observatory. Patient safety core indicator survey response, by country [Internet]. Available from: https://www.who.int/data/gho/data/indicators/indicator-details/GHO/patient-safety-action-plan-core-indicator-survey-response

[33] Kringos DS, Sunol R, Wagner C, Mannion R, Michel P, Klazinga NS, et al. The influence of context on the effectiveness of hospital quality improvement strategies: a review of systematic reviews. BMC Health Serv Res [Internet]. 2015;15(1):277. Available from: 10.1186/s12913-015-0906-0 10.1186/s12913-015-0906-0 PMC4508989 26199147

[34] Li SA, Jeffs L, Barwick M, Stevens B. Organizational contextual features that influence the implementation of evidence-based practices across healthcare settings: a systematic integrative review. Syst Rev [Internet]. 2018;7(1):72. Available from: 10.1186/s13643-018-0734-5 10.1186/s13643-018-0734-5 PMC5936626 29729669

[35] Shea CM, Turner K, Albritton J, Reiter KL. Contextual factors that influence quality improvement implementation in primary care: the role of organizations, teams, and individuals. Health Care Manage Rev. 2018;43(3):261–9. 29533271 10.1097/HMR.0000000000000194 PMC5976517

[36] Cope EL, Johnson M, Khan M, Kaplan HC, Sales A, Mistry KB. Contextual factors affecting implementation of pediatric quality improvement programs. Acad Pediatr. 2022;22(3S):S81–91. 35339248 10.1016/j.acap.2021.08.016

[37] World Health Organization. Global strategy on human resources for health: workforce 2030 [Internet]. World Health Organization; 2016. Available from: https://iris.who.int/handle/10665/250368

