# Supplementary material for: Understanding the development and implementation of national quality of care and patient safety strategic documents: a scoping review
Source: BMC Health Serv Res. 2025 Nov 27;25:1546. doi: 10.1186/s12913-025-13563-2 (PMC12681144; doi:10.1186/s12913-025-13563-2)
Supplement: Supplementary file 2 — Supplementary Material 2: Information on the development process mentioned in the 12 articles [file 12913_2025_13563_MOESM2_ESM.docx]

Additional file 1 – Information on the development process mentioned in the 12 articles

| **Article** | **Stakeholders involved in the design process** | **Priorities and/or Areas of Focus and/or Goals of the initiative** |
| --- | --- | --- |
| National patient safety consortium: learning from large-scale collaboration | Establishment of a National Patient Safety Consortium to drive a shared action plan for safer healthcare. The Consortium had 50 organizations from across Canada - governments (federal, provincial and territorial); professional groups; patient and Families; health service delivery organizations; regulators; educators and national and provincial quality and safety organizations and comittees; pan-Canadian organizations | Aras of focus: surgical care safety, medication safety, infection prevention and control and homecare safety. Patient safety education identified as a foundational underpinning to advancing patient safety improvement in all of these areas. |
| A study of the implementation of patient safety policies in the NHS in England since 2000: what can we learn? | Not defined | Not defined |
| The Better Care Plan: A blueprint for improving America’s healthcare system | Not defined | three main actions: to reorganize care to be continuously improving and patient-centered; to expand risk-adjusted prospective payments; and to make publicly available patient safety and outcome quality-of-care data Seven design principles:  - To change how care is organized and deliver, there should be: 1. integrated and coordinated team-based, technology enabled, patient-centered primary care; 2. continuous improvement in care; 3. continuous efforts to eliminate inequities in care.  - To change how to pay for care, there should be: 4. risk-adjusted prospective payment to provider organizations.  - To change how patient safety and outcomes of care are reported, there should be: 5. patient access to personal health records and information on plan/provider organization performance; 6. transparency and accountability of health system patient safety and quality-of-care outcome performance measures for use by consumers, purchasers, and those held accountable for continuously improving care.  - To recognize competitive forces in the US healthcare markets 7. competition should be based on patient safety and quality, access, and price. |
| From accreditation to quality improvement-The Danish National Quality Programme. | Not defined | Aim: strengthen the focus on continuous quality improvement 8 Goals: Better continuity of patient care in clinical pathways; Stronger measures for chronically ill and elderly patients; Higher survival rate and improved patient safety; High quality treatment; Quick assessment and treatment; Greater patient involvement; Additional healthy life years; More efficient healthcare system |
| A Quality Strategy to Advance the Triple Aim in California’s Medicaid Program | To provide thought leadership, technical assistance, consultation, and training to advance the Quality Strategy, the California Department of Health Care Services (DHCS) contracted with the Institute for Population Health Improvement (IPHI), University of California, Davis, because of its demonstrated expertise in Quality improvement and health system transformation. The draft of the Quality Strategy was presented to DHCS leadership and staff and a statewide stakeholder workgroup. More than 5000 internal and external stakeholders. | Goals: Improve the health of all Californians; Enhance quality, including the patient care experience, in all DHCS programs; Reduce the Department’s per capita health care program costs Priorities: Improve patient safety; Deliver effective, efficient, affordable care; Engage persons and families in their health; Enhance communication and coordination of care; Advance prevention; Foster healthy communities; Eliminate health disparities |
| Understanding the factors influencing implementation of a new national patient safety policy in England: Lessons from 'learning from deaths'. | Not defined | Establish a system to gather relevant information on deaths; Synthesise learning across the organisation; Ensure organisation-wide learning and assurance, with transparent reporting on performance; Improve experience for families; Promote inter-organisational learning across care boundaries |
| Quality improvement lessons learned from National Implementation of the "Patient Safety Events in Community Care: Reporting, Investigation, and Improvement Guidebook". | Not defined | Planning for implementation; Engaging key collaborators; Available resources; Networks and communications; Culture; Executing implementation; External policies; Cosmopolitanism; |
| The Danish health care quality programme: Creating change through the use of quality improvement collaboratives. | Not defined | (1) specialised palliation, (2) stroke treatment, (3) treatment for aged patients ≥65 years old with upper femur hip fractures and, (4) use of antibiotics in hospitals. Additional QICs are planned or currently implemented, including child diabetes, and chronic obstructive pulmonary disease. |
| Reporting and use of the OECD Health Care Quality Indicators at national and regional level in 15 countries | Not defined | Quality indicators |
| Quality improvement and accountability in the Danish health care system | Not defined | Reduction of documentation, ensuring equal access to everyone, free of charge and self-determined |
| Effects of the Italian Law on Patient Safety and Health Professional Responsibilities Five Years after Its Approval by the Italian Parliament | Not defined | Ensure that every person has the right to a safe care, protection of health professionals, transparency of the processes and outcomes and fair compensation in the event of harm |
| Analysing 'big picture' policy reform mechanisms: the Australian health service safety and quality accreditation scheme | Stakeholder consultation took 5 years. During this time, stakeholders were consulted to gain input to the review, using diverse consultation mechanisms. Regulators, accreditation agencies, insurers, health services, research experts and consumers were consulted | Improve the quality of care and safety of patients |
