# Supplementary material for: Understanding the development and implementation of national quality of care and patient safety strategic documents: a scoping review
Source: BMC Health Serv Res. 2025 Nov 27;25:1546. doi: 10.1186/s12913-025-13563-2 (PMC12681144; doi:10.1186/s12913-025-13563-2)
Supplement: Supplementary file 3 — Supplementary Material 3: Information on the development process of the 17 analysed documents [file 12913_2025_13563_MOESM3_ESM.docx]

Additional file 2 - Information on the development process of the 17 analysed documents

| **Document** | **Stakeholders involved** | **Consultation process** | **Identified Priorities** |
| --- | --- | --- | --- |
| Patient safety strategy 2.0 | Patient Safety Advisory Board and specialist group | Not defined | 1. Policy development  2. Organizational development  3. Human resources/personnel development  4. Citizens and patients in Austria |
| Quality strategy for the Austrian healthcare system Version 2.1 | Not defined | Not defined | Topic focus and themes: 1. Patient safety: 1.1. Patient safety strategy and 1.2 hygiene 2. Quality measurement: 2.1. Quality measurement in the inpatient area (A-IQI), 2.2. Quality measurement in the outpatient sector and 2.3. Regular cross-sector meetings, patient surveys and participation in the Austrian Health Survey (ATHIS) 3. Quality management: 3.1. Minimum requirements for quality management; 3.2. Further education and training  4. Quality standards and integrated care: 4.1. Quality standards (QS); 4.2 integrated care (IV) 5. Evidence based: 5.1. Evidence-based medicine (EbM), Health Technology Assessment (HTA) and evidence based Public Health (EbPH) 6. Transparency and information: 6.1. Quality reporting; 6.2. Clinic search, hospital compass and rehabilitation compass |
| The Client and Patient Safety Strategy and Implementation Plan 2022–2026 | The preparation of the objectives of the Client and Patient Safety Strategy 2022–2026 began with an eight-part series of workshops organised between May and August 2021 by the Ministry of Social Affairs and Health and implemented by the Vaasa Hospital District’s Finnish Centre for Patient and Client Safety Improvement. The workshops were attended by representatives from central government and public authorities, healthcare and social welfare professionals and developers, representatives of clients and patients, and political decision-makers. On average, each workshop drew more than a hundred participants. Strategy preparations drew on the expertise of a wide variety of stakeholders through discussions and consultation rounds. Experts involved in the preparatory phase represented the Ministry of Education and Culture, the Finnish Institute for Health and Welfare, the Finnish Medicines Agency Fimea, the National Supervisory Authority for Welfare and Health, the Regional State Administrative Agencies, the National Advisory Board on Social Welfare and Health Care Ethics, the Finnish Institute of Occupational Health, the National Medication Safety Network, the National Medical Device Safety Network, the National Health Care Network of Universities of Applied Sciences, and universities, among other parties. | Yes | Strategic priorities: 1. Together with clients and patients 2. Thriving and competent professionals 3. Safety first in all organisations 4. Enhanced best practices 5. cross-cutting theme - collaboration and development of networking. Strengthen and create conditions for national and regional client and patient safety work. |
| National Patient Safety Programme | The National Patient Safety Programme is a cross-disciplinary project. All the players in the healthcare system are represented on its steering committee. | Yes | The project is structured around four themes: Axis 1: strengthening patient information and partnership in the healthcare provider-patient relationship, to enable patients to be co-players in their own safety = Patient information, the patient as co-actor in his or her own safety. Axis 2: setting up a system for reporting and taking into account undesirable events associated with care, with a view to learning and improving practices by analysing the causes systemic and team feedback, with regional and national valorisation. Axis 3: to improve the safety culture: introducing training in healthcare safety into the curriculum of all healthcare professionals and making it a priority in Continuing Professional Development (CPD) programmes; using innovative teaching methods (healthcare simulation); setting up regional support structures for healthcare quality and safety to assist healthcare professionals, as part of the organisations defined by the regional health agencies (RHA). Axis 4: development of research into the safety of healthcare involving disciplines that interact with health (social sciences, economics) and improving the safety of people taking part in clinical research |
| 1^st^ roadmap 2023-2025 “Improving patient safety and residents”. A continuation of the national patient safety program 2013-2017 | In February 2023, the general management of healthcare provision validated the establishment of a steering committee responsible for designing and monitoring the roadmap. This committee is made up of HAS , AFDS , FORAP , France Assos Santé , representatives of ARS, DGS and DGOS. Last June, the members of the steering committee validated the structuring of the 2023-2025 roadmap “Improving the safety of patients and residents” as well as the actions that comprise it. | Yes | Ambition - strengthen the safety culture with the aim of reducing the occurrence of adverse events associated with care that are known to be avoidable  Priorities: develop safety culture and improve patient/resident safety |
| Patient Safety Strategy 2019-2024 | A Co-design Group was established to develop the Patient Safety Strategy. The group had representation from patients and both corporate and service provision levels of the health service. This Patient Safety Strategy was developed by patients and staff. | Yes | Vision: All patients engaging with our health and social care services will consistently receive the safest care possible.  Objective: To improve the safety of all patients by identifying and reducing preventable harm within the health and social care system. |
| National Plan for Patient Safety 2021 - 2026 | Central Healthcare system administration; regional administrations; National medicines authority; Universities; Health ministry | Yes | To consolidate and promote safety on healthcare |
| National Strategy for Health Quality 2015-2020 | Health Directorate; Gulbenkian Foundation | Yes | a) Focus on local interventions, services, provider units and institutions b) Improving clinical and organizational quality; c) Increased adherence to clinical guidelines; d) Strengthening patient safety; e) Strengthening clinical research; f) Permanent monitoring of quality and safety; g) Dissemination of comparable performance data; h) Recognition of the quality of health units; i) Transparent information to citizens and increasing their empowerment. |
| Patient Safety Strategy | Not defined | Not defined | 1. Establish five key patient safety goals; 2. Select specific activities to be implemented to achieve these goals; 3. Determine a timeframe for the implementation of these activities (for AZUS and healthcare facilities); 4. Prepare educational materials to describe specific activities and expectations for implementation; 5. Educate the health care system, especially those facilities participating in accreditation, regarding the AZUS requirements for patient safety; 6. Monitor the implementation of patient safety requirements; 7. Report on the progress and results of the implementation of patient safety requirements. |
| National Strategy for Patient Safety in Healthcare (2023 - 2031) | Ministry of Health and the Government; Independent National Authority for Quality and Safety in Healthcare; Public and Private Health Service Providers; Professional Chambers and Associations, Civil Society, and Patient Associations and Healthcare Education Providers. | Yes | 1. Ensure strategic and legislative changes; 2. Promote high-quality and safe treatment methods with a fair system of financing; 3. Use all the resources optimally and rationally; 4. Empower patients |
| Patient Safety Strategy for the National Health System 2015-2020 | Ministry of Health, Social Services and Equality; Instituto Nacional de Gestión Sanitaria (INGESA); Autonomous Community administrations | Yes | Strategic lines of action: 1. Patient safety culture, human and organizational factors and training. 2. Safe clinical practices. 3. Risk management and reporting and learning systems. 4. Patient and caregiver participation for their safety. 5. Research into patient safety. 6. International Participation. |
| National Action Plan for Increased Patient Safety in Swedish Health Care 2020-2024: Act for safer healthcare | The Action Plan is developed in broad cooperation with authorities, representatives from principals and national organisations, representatives of private caregivers, patients and relatives, experts and students and other stakeholders in the area of patient safety. | Yes | The vision for our joint patient safety work is: Good and safe care – everywhere and always. The overall goal is that: No patient should have to suffer an avoidable injury  Five priority focus areas: 1. knowledge of adverse events; 2. Reliable and safe systems and processes; 3. Safe care; 4. learning and development and 5. risk awareness and preparedness |
| The NHS Patient Safey Strategy | National patient safety teams; NHS England; NHS Improvement regional teams | Yes | Strategy based on two foundations: Creation of a patient safety culture and a Patient Safety System across all settings of care. To do so three strategic aims we developed: 1. improving understanding of safety by drawing intelligence from multiple sources of patient safety information (Insight); 2. equipping patients, staff and partners with the skills and opportunities to improve patient safety throughout the whole system (Involvement); 3. designing and supporting programmes that deliver effective and sustainable change in the most important areas (Improvement). |
| National Safety and Quality Health Service Standards - 2nd edition | This document includes the views or recommendations of its authors and third parties. The National Safety and Quality Health Service (NSQHS) Standards were developed by the Australian Commission on Safety and Quality in Health Care (the Commission) in collaboration with the Australian Government, states and territories, the private sector, clinical experts, patients and carers. | Yes | The primary aims of the NSQHS Standards are to protect the public from harm and to improve the quality of health service provision.  There are eight NSQHS Standards (main areas) 1) Clinical Governance: which describes the clinical governance, and safety and quality systems that are required to maintain and improve the reliability, safety and quality of health care, and improve health outcomes for patients 2) Partnering with Consumers: which describes the systems and strategies to create a person-centred health system by including patients in shared decision making, to ensure that patients are partners in their own care, and that consumers are involved in the development and design of quality health care 3) Preventing and Controlling Infections: which describes the systems and strategies to prevent infection, manage infections effectively when they occur, limit the development of antimicrobial resistance through prudent use of antimicrobials (as part of effective antimicrobial stewardship), and promote appropriate and sustainable use of infection prevention and control resources 4) Medication Safety: which describes the systems and strategies to ensure that clinicians safely prescribe, dispense and administer appropriate medicines to informed patients, and monitor use of the medicines 5) Comprehensive Care: which describes the integrated screening, assessment and risk identification processes for developing na individualised care plan, to prevent and minimize the risks of harm in identified areas 6) Communicating for Safety: which describes the systems and strategies for effective communication between patients, carers and families, multidisciplinary teams and clinicians, and across the health servisse organisation 7) Blood Management: which describes the systems and strategies for the safe, appropriate, efficient and effective care of patients’ own blood, as well as other supplies of blood and blood products 8) Recognising and Responding to Acute Deterioration: which describes the systems and processes to respond effectively to patients when their physical, mental or cognitive condition deteriorates  which cover: a) high-prevalence adverse events, b) preventing and controlling infections, c) medication safety, d) comprehensive care, e) clinical communication, f) the prevention and management of pressure injuries, g) the prevention of falls, h) responding to clinical deterioration |
| Improving safety and quality in health care - A strategic plan for action in WA 2024-2026 | Extensive engagement with approximately 148 consumers, carers and/or family members, 390 healthcare staff, 90 Department of Health staff and 78 health executives and board members told us what the safety and quality priorities for WA Health in the next 3 years should be and how their accomplishment can be supported. The final draft of the WA safety and quality strategic plan was also reviewed by a range of people to ensure it was clear and the direction and priorities for safety and quality in WA Health were ambitious but achievable | Yes | AIM: Safer, higher-performing and more person-centred health care for all Western Australians by June 2026  Priorities: Safe; High-performing; Person-centred |
| The Canadian Quality and Patient Safety Framework for Health Services | Health teams (patients included); Health leaders; Board members; Policy makers | Yes | Goal 1: Provide people-centred care. Goal 2: Provide safe care. Goal 3: Provide accessible care. Goal 4: Provide appropriate care. Goal 5: Provide integrated care |
| Safer Together: A National Action Plan to Advance Patient Safety | The Institute for Healthcare Improvement convened the National Steering Committee for Patient Safety as a collaboration among 27 national organizations committed to advancing patient safety. Organizations and individual members contributed to develop the National Action Plan to Advance Patient Safety and have committed to advancing the recommendations outlined. Membership in the NSC is voluntary and includes leaders from 27 organizations representing the following groups: Health care organizations and health care systems; Patients, families, and care partners; Professional societies; Safety and quality organizations; Regulatory and accrediting bodies; Federal agencies. | Yes | Four foundational and interdependent areas: 1. Culture, Leadership, and Governance: The imperative for leaders, governance bodies, and policymakers to demonstrate and foster our deeply held professional commitments to safety as a core value and promote the development of cultures of safety. 2. Patient and Family Engagement: The spread of authentic patient and family engagement; the practice of co-designing and co-producing care with patients, families, and care partners to ensure their meaningful partnership in all aspects of care design, delivery, and operations. 3. Workforce Safety: Ensuring the safety and resiliency of the organization and the workforce is a necessary precondition to advancing patient safety; we need to work toward a unified, total systems-based perspective and approach to eliminate harm to both patients and the workforce. 4. Learning System: Establishing networked and continuous learning; forging learning systems within and across health care organizations at the local, regional, and national levels to encourage widespread sharing, learning, and improvement.  Three cross-cutting themes that are integral to the four foundational areas and recommendations in the National Action Plan: 1. Person-centered care: Patients, families, and care partners experience lifelong care journeys across the entire continuum of care. They have a unique and essential perspective on care delivery, and their insights on “what matters” are critical for creating safer care. 2. Care across the entire continuum: Care is provided in locations outside of hospitals, including ambulatory, long-term care facilities, home, and other community-based settings. The recommendations in this National Action Plan are meant to be relevant to all settings across the care continuum. 3. The relationship between patient safety and health equity: Health inequities are “linked to the complicated history and reality of racism, classism, sexism, ableism, ageism, and other forms of oppression.” Inequities result in a concentration of harm in specific population groups, based on characteristics such as race, ethnicity, sexual orientation, gender, age, disability, and income and must be considered when designing safety efforts to ensure that inequities are being addressed. |
