# Supplementary material for: Understanding the development and implementation of national quality of care and patient safety strategic documents: a scoping review
Source: BMC Health Serv Res. 2025 Nov 27;25:1546. doi: 10.1186/s12913-025-13563-2 (PMC12681144; doi:10.1186/s12913-025-13563-2)
Supplement: Supplementary file 4 — Supplementary Material 4: Information on the development process of the 17 analysed documents (main objectives) [file 12913_2025_13563_MOESM4_ESM.docx]

Additional file 3 - Information on the development process of the 17 analysed documents (main objectives)

| **Document** | **Main Objectives** | | | | | | | |
| --- | --- | --- | --- | --- | --- | --- | --- | --- |
|  | **Safety culture** | **Medication Safety** | **Communication** | **Safe Surgery** | **Healthcare associated infections** | **Patient engagement** | **Patient identification** | **Other** |
| Patient safety strategy 2.0 | Organizational development: continuous improvements in patient safety;  Human resources/personnel development: continuous expansion and improvement of competencies on the subject of patient safety | Not applicable | Not applicable | Not applicable | Not applicable | Citizens and patients in Austria: increase the population's competence on the subject of patient safety. | Not applicable | Policy development: further development of the strategy and patient safety |
| Quality strategy for the Austrian healthcare system Version 2.1 | Not applicable (it's a quality strategy) | Not applicable (it's a quality strategy) | Not applicable (it's a quality strategy) | Not applicable (it's a quality strategy) | Not applicable (it's a quality strategy) | Not applicable (it's a quality strategy) | Not applicable (it's a quality strategy) | Main goals for each topic focus 1.1 Ensuring/guaranteeing the safety of patients in the Austrian healthcare system; 1.2 Prevention of healthcare system-associated infections and antimicrobial resistance; 2.1 Ensuring the quality of processes and results in the inpatient area in accordance with the best available evidence, continuously promoting quality development; 2.2 Ensuring the quality of processes and results in the entire outpatient area, especially in terms of integrated care; 2.3 Ensuring a high level of satisfaction among patients and the population with the care; 3.1 The minimum requirements for quality management are implemented by all health service providers; 3.2 Ensuring a high level of qualifications for all healthcare professionals, particularly with regard to quality management and new forms of care; 4.1 Ensuring quality-assured care for patients in the Austrian healthcare system based on evidence-based, nationwide quality standards; 4.2 Ensuring integrated health and health care for the population at a high quality level; 5. Using the best available evidence as a basis for rational decisions in the healthcare system; 6.1 Recognizing potential for improvement in quality work in healthcare facilities as well as developing and initiate improvement measures. Continuous development of nationwide and cross-sector reporting on quality in the healthcare system 6.2 Supporting the population (empowerment) in decision-making and preparing for a hospital stay by providing information on neutral platforms |
| The Client and Patient Safety Strategy and Implementation Plan 2022–2026 | Objective 1.2: Promote client safety and patient safety side by side  Objective 2.1: Ensure safety competence and its development throughout careers   Objective 2.2: Create safety by supporting wellbeing at work  Objective 2.3: Improve safety through active leadership  Objective 3.3 Safety culture is the foundation of our daily work | Objective 4.1: Increase medication safety through common practices | Not applicable | Not applicable | Objective 4.3: Harmonise good practices in infection prevention and control | Objective 1.1: Increase engagement to improve safety  Objective 1.3: Use experiences of clients, patients and close ones to guide our service development | Not applicable | Objective 3.1: Use open data and information to guide our actions and increase safety  Objective 3.2: Ensure safe remote and digital services  Objective 4.2: Ensure the safe use of medical devices and information systems |
| National Patient Safety Programme | Axis 3 - Training, safety culture, support General objective 1: Using feedback as the basis for learning about safety General objective 2: Relying on multi-professionality and the "team" to build a culture of safety General objective 3: Mobilising management around safety issues General objective 4: Strengthening the role of healthcare safety in professional training General objective 5: Providing support to all professionals in carrying out their quality and care safety projects | Not applicable | Not applicable | Not applicable | Not applicable | Axis 1 - Patient information, the patient as co-actor in his or her own safety General objective 1: Strengthening the partnership in the care-giver-patient relationship General objective 2: Better patient information General objective 3: Making it easier for user representatives to carry out their duties | Not applicable | Axis 2 - Improving the reporting and handling of adverse events associated with healthcare General objective 1: Improving systems for reporting and declaring adverse events associated with healthcare General objective 2: Promoting the implementation of corrective actions at local level General objective 3: Making the most of data from reporting systems and implementing security solutions General objective 4: Integrate the analysis of the causes of SAEs into existing systems for improving the quality and safety of care  Axis 4 - Innovation and research; Theme 1: Research into patient safety during care General objective 1: Developing research into patient safety Axis 4 - Innovation and research; Theme 2: Patient safety in clinical trials General objective 1: Identifying and dealing with safety risks for patients and volunteers enrolled in clinical trials General objective 2: Improving training for investigators and clinical research project leaders General objective 3: Promoting education on the Act of 5 March 2012 for professionals and users |
| 1^st^ roadmap 2023-2025 “Improving patient safety and residents”. A continuation of the national patient safety program 2013-2017 | 2 transversal objectives to develop safety culture: Tranversal objective 1 - Communicate with field professionals and users Tranversal objective 2 - Train professionals and patients in safety culture | Not applicable | Not applicable | Not applicable | Not applicable | Not applicable | Not applicable | 5 main areas of work to improve patient/resident safety: Axis 1 - Promote and support teamwork and collective time throughout the patient journey Axis 2 - Acting on the under-reporting of serious adverse events associated with care (SAEs) Axis 3 - Improve capitalization on feedback Axis 4 - Pursue targeted actions in certain sectors Axis 5 - Promote the place of the patient and their loved ones to improve safety |
| Patient Safety Strategy 2019-2024 | Ambitions for Patient Safety:  b) for staff: Staff have the information, knowledge, skills, environment, equipment, time and supports required to do their job, to work effectively with others for safety, to improve safety and to identify, implement and sustain new safety practices. c) for systems: We have resilient and safe systems where staff are supported to do their jobs safely and to work together effectively. There is coproduction of safe healthcare with patients and a culture of meaningful measurement and improvement for safety. d) for organisational learning: We have a culture of patient safety which actively promotes, captures, shares, spreads and implements learning to improve patient safety at every level of the organisation | Not applicable | Not applicable | Not applicable | Not applicable | Ambitions for Patient Safety:  a) for patients: Patients have the information, knowledge, skills and supports that they need to feel safe, to take responsibility for their own safety, to contribute to improvements in patient safety and to partner with health and social care services to inform and influence the future development of safe and person-centred care. | Not applicable | Not applicable |
| National Plan for Patient Safety 2021 - 2026 | Promote healthcare worker's education on patient safety; Evaluate safety culture. | Not applicable | Optimize inter and intra institutional communication;   Adjust communication and safety on care transitions;  Adjust clinical information communication for patients, families and carers. | Not applicable | Reduce healthcare associated infections and antimicrobial resistance | Increase health literacy and participation on patient safety for patients, families, carers and society. | Not applicable | 1. Ensure healthcare managment engament on this plan implementation; 2. Consolidate communication between local, regional and national level managment on patient safety; 3. Increase repport culture and transparency on patient safety events (on a repport platform); 4. Promote follow up and evaluation on patient safety events; 5. Consolidate and promote safe practices on healthcare contexts; 6. Monitor safe practices implementation; 7. Promote safe telehealth. |
| National Strategy for Health Quality 2015-2020 | Not applicable (it's a quality strategy) | Not applicable (it's a quality strategy) | Not applicable (it's a quality strategy) | Not applicable (it's a quality strategy) | Not applicable (it's a quality strategy) | Not applicable (it's a quality strategy) | Not applicable (it's a quality strategy) | Not defined (No main objectives were identified) |
| Patient Safety Strategy | Goal 5: Safe care by elimination/minimization of adverse events | Goal 3: Safe medication practices | Not applicable | Goal 1: Safe surgical procedures | Goal 2: Minimal facility-caused infection. | Not applicable | Goal 4: Care and treatment to the right patient | Not applicable |
| National Strategy for Patient Safety in Healthcare (2023 - 2031) | Not defined (No main objectives were identified) | Not defined (No main objectives were identified) | Not defined (No main objectives were identified) | Not defined (No main objectives were identified) | Not defined (No main objectives were identified) | Not defined (No main objectives were identified) | Not defined (No main objectives were identified) | Not defined (No main objectives were identified) |
| Patient Safety Strategy for the National Health System 2015-2020 | General objective 1: Further enhance patient safety culture, human and organizational factors; General objective 2.4: Promote the implementation of safe practices in patient care; General objective 2.7: Promote the design and development of strategies for dealing with severe adverse events at healthcare centers; General objective 3.1: Promote risk management at the medical services centers; General objective 3.2: Promote the implementation and development of systems for reporting health care-related incidents for purposes of learning; General objective 5.1: Promote the further enhancement of the knowledge in the prevent of the harm associated with health care; | General objective 2.1: Promote safe medication use; | General objective 2.6: Promote communication among professionals; | General objective 2.3: Promote the implementation of safe practices in surgery; | General objective 2.2: Promote safe practices for preventing and controlling health care-associated infections; | General objective 4.1: Promote the participation of the patients and their caregivers in patient safety; | General objective 2.5: Promote safer patient identification; | General objective 2.8: Promote the safe use of ionizing radiation in clinical procedures; General objective 6.1: Promote international collaboration in patient safety; |
| National Action Plan for Increased Patient Safety in Swedish Health Care 2020-2024: Act for safer healthcare | Not applicable | Not applicable | Not applicable | Not applicable | Not applicable | Not applicable | Not applicable | 1. Increase knowledge of adverse events; 2. Reliable and safe systems and processes; 3. Safe care here and now; 4. Strengthen analysis, learning and development; 5. Increase risk awareness and preparedness |
| The NHS Patient Safey Strategy | 1. " use existing culture metrics like those in the NHS Staff Survey to understand their safety culture and focus on staff perceptions of the fairness and effectiveness of incident management"; 2. "focus on the development and maintenance of a just culture by adopting the NHS Just Culture Guide or equivalent"; 3. "embed the principles of a safety culture within and across local system organisations, and align those efforts with work to ensure organisations adhere to the well-led framework and its eight key lines of enquiry." | The issue is approached on the aim "Involvement" of this strategy. On the 2021 update, one of the objetives is to deliver the Medication Safety Improvement Programme (MSIP) | Reactive to incident reporting | Reactive to incident reporting | The issue is approached on the aim "Involvement" of this strategy. On the 2021 update, one of the objetives is to deliver the UK National Action Plan for AMR | The issue is approached on the aim "Involvement" of this strategy. On the 2021 update, one of the objetives is to involve patient in patient safety* | Reactive to incident reporting | Reactive to incident reporting |
| National Safety and Quality Health Service Standards - 2nd edition | 1. Clinical Governance Main objective: To implement a clinical governance framework that ensures that patients and consumers receive safe and high-quality health care | 4. Medication Safety Main objective 1: To ensure clinicians are competent to safely prescribe, dispense and administer appropriate medicines and to monitor medicine use.  Main objective 2:To ensure consumers are informed about medicines and understand their individual medicine needs and risks. | 6. Communicating for Safety Main objective: To ensure timely, purpose-driven and effective communication and documentation that support continuous, coordinated and safe care for patients. | Not applicable | 3. Preventing and Controlling Infections Main objective 1: To reduce the risk to patients, consumers and members of the workforce of acquiring preventable infections Main objective 2: Effectively manage infections, if they occur. Main objective 3: Prevent and contain antimicrobial resistance Main objective 4: Promote appropriate prescribing and use of antimicrobials as part of antimicrobial stewardship Main objective 5: promote appropriate and sustainable use of infection prevention and control resources | 2. Partnering with Consumers Main objective: To create an organisation in which there are mutually valuable outcomes by having: a) Consumers as partners in planning, design, delivery, measurement and evaluation of systems and services; b) Patients as partners in their own care, to the extent that they choose. | Not applicable | 5. Comprehensive Care Main objective 1: To ensure that patients receive comprehensive care – that is, coordinated delivery of the total health care required or requested by a patient. This care is aligned with the patient’s expressed goals of care and healthcare needs, considers the effect of the patient’s health issues on their life and wellbeing, and is clinically appropriate Main objective 2: To ensure that risks of harm for patients during health care are prevented and managed. Clinicians identify patients at risk of specific harm during health care by applying the screening and assessment processes required in this standard.  7. Blood Management Main objective: To identify risks, and put in place strategies, to ensure that a patient’s own blood is optimised and conserved, and that any blood and blood products the patient receives are appropriate and safe.  8. Recognising and Responding to Acute Deterioration Main objective: To ensure that a person’s acute deterioration is recognised promptly, and appropriate action is taken. Acute deterioration includes physiological changes, as well as acute changes in cognition and mental state. |
| Improving safety and quality in health care - A strategic plan for action in WA 2024-2026 | Main objective 1: Safer care for patients, supported by a compassionate workforce with a strong safety culture. | Not applicable | Not applicable | Not applicable | Not applicable | Main objective 3: Actively partner with consumers and clinicians to recognise and respond to the evolving and diverse needs of the community | Not applicable | Main objective 2: Establish and embed health care infrastructure to create a sustainable systemwide focus on quality planning, quality improvement and quality control |
| The Canadian Quality and Patient Safety Framework for Health Services | Not defined (No main objectives were identified) | Not defined (No main objectives were identified) | Not defined (No main objectives were identified) | Not defined (No main objectives were identified) | Not defined (No main objectives were identified) | Not defined (No main objectives were identified) | Not defined (No main objectives were identified) | Not defined (No main objectives were identified) |
| Safer Together: A National Action Plan to Advance Patient Safety | Main Aim 1. On Culture, Leadership, and Governance: Health care organization governing boards and CEOs across the care continuum (healthcare leaders) establish and sustain a strong culture of safety in a way that is equitable and engaging of patients, families, care partners, and the health care workforce. Main Aim 3. On Workforce Safety: Health care organizations across the care continuum implement strategies to measurably and equitably improve safety for health care professionals and all staff in their organizations. | Not applicable | Not applicable | Not applicable | Not applicable | Main Aim 2. On Patient and Family Engagement: Health care organizations institute strategies to improve safety, as defined by patients, families, care partners, and the workforce, in all settings across the care continuum. | Not applicable | Main Aim 4. On Learning System: Health care organizations and other stakeholders across the care continuum implement reliable learning systems. These learning systems actively engage with local, regional, state, or national learning systems to develop a national learning network of existing and future learning systems. |
