# Supplementary material for: Understanding the development and implementation of national quality of care and patient safety strategic documents: a scoping review
Source: BMC Health Serv Res. 2025 Nov 27;25:1546. doi: 10.1186/s12913-025-13563-2 (PMC12681144; doi:10.1186/s12913-025-13563-2)
Supplement: Supplementary file 5 — Supplementary Material 5: Information on the implementation process mentioned in the 12 articles [file 12913_2025_13563_MOESM5_ESM.docx]

Additional file 4 - Information on the implementation process mentioned in the 12 articles

| **Article** | **Implementation Process** |
| --- | --- |
| National patient safety consortium: learning from large-scale collaboration | - The governance model was put in place. CPSI served as the coordinating body, a steering committee was established, an advisory communications group began implementing a strategic communications plan and an evaluation action team developed a logic model and evaluation plan. Leads groups for each area of focus were established to review progress, barriers and enablers to advancing the respective action plans. Action teams were struck to operationalize individual actions. Patients were engaged as full partners at all levels of governance. The structure was a shared leadership model focusing on collaboration, with patients and families participating at all levels. |
| A study of the implementation of patient safety policies in the NHS in England since 2000: what can we learn? | Core themes that represent the conditions/factors required for the effective implementation of patient safety policy initiative (there are close inter-relationships between the themes identified):  - Capability - Ensuring support for building patient safety capability: Ensuring healthcare workers have the skills and tools to properly investigate, learn from and address patient safety incidents. This includes training and methods for improving safety practices. The concept of human factors is also important, focusing on the interaction between people and systems, as well as improving teamwork and communication. This theme emphasizes the importance of systematically learning from incidents and using those lessons to improve safety processes.  - Culture - Ensuring a shift in patient safety culture: Changing the culture within healthcare is necessary to improve patient safety. Shifting towards a culture of "no blame", where staff can report incidents without fear of punishment and learn from them. This would encourage more reporting of errors and lead to better learning from mistakes. This is key to fostering continuous improvement and transparency. Focusing on positive cultural change and improving engagement with staff will foster continuous improvements in patient safety.  - Systems - Ensuring a systems approach and systems thinking: Creating strong systems for managing patient safety. This involves having the right processes, tools, and environments in place to prevent errors and respond effectively when they occur. The article notes that many patient safety incidents are due to systems failures, and policies should focus on fixing those underlying issues rather than blaming individuals. The article also points out that improving incident reporting systems is essential for learning from errors and making healthcare safer.  - Candour - Promoting candour with patients and families: Promoting openness and honesty with patients and their families when things go wrong, ensuring trust and accountability in healthcare settings. The article highlights that being transparent about errors helps build trust and can lead to patients being more accepting of mistakes. The legal duty of candour, introduced after the Mid Staffordshire NHS scandal, requires hospitals to inform patients when incidents occur and to apologize for them. This regulation aims to make healthcare more transparent and ensure that organizations are accountable for patient safety.  - Leadership - Ensuring strong leadership: Strong leadership is crucial to drive patient safety improvements. Leaders need to be engaged and committed to fostering a culture of safety and supporting long-term strategies. The article notes that strong clinical leadership and board engagement are necessary to ensure that safety becomes a top priority in healthcare organizations.  Sub-themes associated with the 5 core themes: 1. Capability - a) Support for building capability in reviewing patient safety incidents, using clear and efficacious methods that systematically enable lessons to be learned; b) Support for building capability in using patient safety resources and improvement techniques to enable the delivery of safety solutions to problems in care 2. Culture - a) Ensuring a shift towards a ‘no blame’ culture, to deliver improvements to patient safety outcomes by supporting the understanding of how systems failures lead to patient safety incidents; b) Ensuring a shift towards an open and fair culture to maximize reporting of incidents and learning from them; c) Ensuring a shift towards an open and fair culture to increase transparency in engaging about risks associated with the delivery of healthcare; d) Ensuring a shift towards positive cultural and behavioural change through staff engagement, to enable continuous improvement. 3. Systems - a) Implementation of effective incident reporting systems as part of approaches to improving patient safety; b) Implementation of co-ordinated, patient safety systems-based approaches to delivering care, underpinned by Strong evidence-based interventions, to increase safety by mitigating unwarranted risks 4. Candour - a) Promotion of candour with patients and families involved in patient safety incidents, as part of an effective patient safety system that is open to learning 5. Leadership - a) Ensuring strong leadership commitment, including clinical leadership and engagement, to facilitate and advance patient safety improvement; b) Ensuring ongoing investment in board development in order to support long-term thinking and strategic, system-wide approaches to patient safety improvement |
| The Better Care Plan: A blueprint for improving America’s healthcare system | Number of challenges to the implementation/adoption of the programme's principles and criteria:  - Health plans, provider organizations, and others have been well rewarded by the longstanding entrenched fee-for-service payment system, encouraging the provision of largely acute and specialist-oriented care.  - The system is reactive and transactional, waiting for patients to arrive for care, not proactive and relational. The system is highly resistant to change. A century of habits instilled in medical and health professional education, practice, financing, and payment needs to be overcome |
| From accreditation to quality improvement-The Danish National Quality Programme. | A one-year educational programme for quality management was developed - the aim was to introduce to and qualify healthcare leaders in improvement methods. Quality improvement collaboratives were designed - the aim was to engage clinical staff in improvement projects and promote a faster distribution of knowledge. The designated areas are characterized by quality problems or undesirable variation. Examples of designated areas are palliative care, early stroke care, rational use of antibiotics and restraint in mental health.   Leadership is considered a major key for the NQP to succeed. The NQP address quality via local involvement and collaboration between all sectors is essential. Hence, in contrast to previous initiatives, the responsibility of the practical implementation of the NQP is primarily placed at a local level. The transition into local initiatives may ensure that the goals and associated quality improvement initiatives are considered more relevant and effective. |
| A Quality Strategy to Advance the Triple Aim in California’s Medicaid Program | Once the draft was complete, the QS team launched the implementation process with 3 concurrent tasks: (1) gather stakeholder input, (2) establish an external advisory committee, and (3) conduct a baseline QI inventory.  1) Stakeholder Input: - The draft QS was presented to DHCS leadership and staff and a statewide stakeholder workgroup. More than 5000 internal and external stakeholders also were invited to a QS webinar. The webinar covered the QS contents and implementation process; Real-time online polling showed overwhelming support for the proposed goals, priorities, and guiding principles. Following the webinar, attendees were sent an electronic survey to collect more nuanced feedback. Responses expressing concern, confusion, or suggestions were handled with follow-up communication to ensure that the participant’s feedback was adequately incorporated into the final draft or to explain why the feedback was not incorporated. 2) External Advisory Committee:   - To obtain ongoing external guidance, the Medi-Cal Performance Advisory Committee (MPAC) was convened. The MPAC met for a full day 7 times between 2012 and 2014. It consisted of a multidisciplinar group of 9 prominent thought leaders from academia, health plans, hospitals, foundations, and local government. The MPAC provided diverse perspectives to help the QS team make practical, evidence-based recommendations to advance QI and navigate systems change within DHCS. 3) Baseline QI Inventory:  - The QS team developed a survey to capture information about DHCS QI activities in clinical care, health promotion and disease prevention, and administration. Under each domain, the survey asked participants to report any QI activities and provide detailed information. Participants also were asked to identify: any quality metrics that were collected but not explicitly linked to any QI activities; perceived gaps in the department’s QI activities; and suggestions for future QI activities. Surveys were sent to all 35 offices and divisions within DHCS as well as to 3 other leaders of special subgroups within the organization.  Throughout the 3 tasks, the QS team worked to build relationships across DHCS.  The QS team learned through the baseline survey and stakeholder outreach that many DHCS division leaders were unfamiliar with Quality Improvement (QI) concepts, such as specific aims and objectives. To clarify, they included definitions in the baseline survey and held personal meetings with leaders to discuss their activities and determine if they qualified as QI. Some divisions were already conducting QI without realizing it, while others collected useful metrics without linking them to QI. The QS team took a supportive and inclusive approach, aiming to understand the current QI efforts without judgment or punitive measures. Instead, they framed QS as an opportunity to showcase or develop innovative programs. Where QI was lacking, it was seen as a chance to implement it. To ensure inclusivity, the QS used a broad definition of QI, including indirect or administrative activities. The initiative was positioned as a way to promote a culture of quality (COQ) and improve health outcomes.  Quality improvement activities were reported and/or developed for each of the priorities identified.  Federal dollars funded many of the QI activities, and DHCS QI activities often paralleled national QI activities.  From the outset, the director of DHCS encouraged transparency and engagement in the QS. Thus, executives, managers, supervisors, and line staff in the department felt free to share both support and concerns for all policies and programs. |
| Understanding the factors influencing implementation of a new national patient safety policy in England: Lessons from 'learning from deaths'. | Interviewees indicated that early progress in the implementation process was bolstered by peer collaboration, facilitated by Academic Health Science Networks (AHSNs)—organizations that bridge the NHS, academic institutions, local authorities, the third sector, and industry. This collaboration fostered the exchange of best practices between organizations and provided valuable feedback on various implementation approaches. However, interviewees also noted that the implementation of LfD was executed rapidly and in a top-down manner. As a result, Trusts had limited time to fully integrate the programme into their existing safety infrastructures, occasionally leading to the development of parallel systems for delivery. The five policymakers goals for implementing the LfD policy were a) explicit case selection and review process that gathers both quantitative and qualitative information b) Greater board involvement c)integrating insights from LfD with other sources of safety information to enhance learning d) mechanisms to gather valuable information from them to contribute to patient safety improvement e) establish systems whereby those organisations come together to examine the quality of that care |
| Quality improvement lessons learned from National Implementation of the "Patient Safety Events in Community Care: Reporting, Investigation, and Improvement Guidebook". | Several ERIC implementation strategy groupings were identified: “Develop collaborator interrelationships,” “Train and educate collaborators,” “Provide interactive assistance,” and “Support clinicians. ”Lesson 1: Engage all collaborators involved in implementation - Engagement with all collaborators involved in the implementation emerged as being one of the most important lessons learned during all phases of implementation. Lesson 2: Ensure end-users have opportunities to provide feedback. Ensuring end-users have opportunities to provide feedback emerged as one of the next most important lessons learned. Lesson 3: Describe collaborators' purpose and roles/responsibilities clearly at the start We found evidence describing the importance of defining collaborators' purpose and roles/responsibilities clearly throughout the planning and implementation phases; Lesson 4: Communicate information widely and repeatedly - Sharing consistent information across multiple levels of an organization; Lesson 5: Identify how multiple high priorities can be synergistic - We found that when collaborators had stronger relationships, Guidebook implementation occurred more smoothly, with staff more actively engaged in working together toward common goals. |
| The Danish health care quality programme: Creating change through the use of quality improvement collaboratives. | a) Introduction of learning sessions about best practices, improvement methods and ideas. The Quality improvement teams are supported by a faculty of clinical experts who identify best practices and facilitate the implementation of improvement initiatives. Throughout the implementation period, which usually lasts about two years, the QI teams receive support at both the national and regional levels. This support includes national learning seminars, training in improvement methods, data for progress evaluation, and meetings with regional coordinators |
| Reporting and use of the OECD Health Care Quality Indicators at national and regional level in 15 countries | Not applicable |
| Quality improvement and accountability in the Danish health care system | Denmark has nationwide health quality improvement initiatives such as: - National clinical guidelines - National surveys on patient experiences - National Agency for Patient's Rights and Complaints and reporting of adverse events - The Danish Health Quality Programme - National Healthcare system monitoring using clinical databases - Public reporting on quality of care "- National clinical guidelines, developed by the Danish Health and Medicines Authority and the Organization of Danish Medical Societies. Disease management programmes and pathway initiatives were also developed aiming at standardizing the delivery of healthcare, ensuring that health practice follows the principles of evidence based medicine, and that medical care is carried out at the same high standard nationwide.  Guidelines are disease or speciality based, and disease management programmes/pathways are anchored in them describing what should be done and by whom. - National surveys on patient experiences: surveys were established in cooperation between the Ministry of health and the Danish regions. The surveys are annual and approach clinical services; patient safety; patient and staff-member continuity; co-involvement and communication; information; course of treatment; discharge and inter-sectorial cooperation. - National Agency for Patient's Rights and Complaints and reporting of adverse events where patients can report an adverse event or file a complaint. Adverse events are reported, collected and analysed on hospital level or at management unit level. The responsible organizations are obliged to analyse adverse events and develop action plans. The Danish Health Quality Programme: it is a national and interdisciplinary accreditation system that emphasizes hospitals, pharmacies, and prehospital units. Standards for accreditation have been developed with representatives from the Danish regions, municipalities and other stakeholders. It is mandatory for hospitals, pharmacies and prehospital units to renew the accreditation every third year.  National Healthcare system monitoring using clinical databases: An organization was created (by several healthcare stakeholders) to improve prevention, diagnostics, treatment, care and rehabilitation, to provide documentation for clinical governance and priority setting, and to create information on the quality of care for patients and consumers of healthcare. Specific clinical process and outcome indicators and quality standards were developed (to each disease/clinical condition) and are helpful to secure efficient data collection. Participation is mandatory for all hospitals, relevant clinical departments and units treating patients with diseases and clinical conditions included in the databases. Data are transmitted electronically and collected in clinical databases.  Public reporting on quality of care: All data and results achieved in the national quality improvement initiatives are available on the Danish e-health portal, that is available to professionals and public, supporting empowerment of patients and giving health professionals better tools for improvement." |
| Effects of the Italian Law on Patient Safety and Health Professional Responsibilities Five Years after Its Approval by the Italian Parliament | The law requires the establishment of a center for healthcare risk management and patient safety in every Italian region, but also: 1. The collection of data on risks and adverse events from public and private healthcare and social facilities (and it's transmission to the national observatory on best practices on patient safety); 2. The appointment of a Clinical Risk Manager in all public and private healthcare facilities; 3. The introduction of guidelines and safe practices recognized by the national institute of health; 4. The creation of a safe environment with the reporting and learning system. The regional centers for healthcare risk management and patient safety manage claims and adverse events. They can also assess the risk of diagnostic and therapeutic inappropriateness in clinical pathways; perform analysis of adverse events and claims; manage reporting and learning systems; train staff on patient safety; implement patient safety practices; support hospital legal departments and prepare annual reports.  A significant limitation to the implementation of the law is that financial investment was not provided (mostly for all the new roles and functions) |
| Analysing 'big picture' policy reform mechanisms: the Australian health service safety and quality accreditation scheme | During the implementation of this accreditation scheme, there were several challenges: 1. Difficulty of developing and maintaining consistent expectations amongst frontline workers requiring the aims and requirements of the reform, 2. Reliably assessing the compliance of institutions with clinical performance measures and their continuous quality improvement, In the other hand, there were some facilitators identified: 1. Regular and diverse consultation activities; 2. Disseminating educative materials that are both informative and easily accessed to different stakeholders; 3. Consistent administration of, and ongoing review and improvements; 4. Outcome data used to inform accreditation operations and ongoing quality improvement actions. |
