# Supplementary material for: Understanding the development and implementation of national quality of care and patient safety strategic documents: a scoping review
Source: BMC Health Serv Res. 2025 Nov 27;25:1546. doi: 10.1186/s12913-025-13563-2 (PMC12681144; doi:10.1186/s12913-025-13563-2)
Supplement: Supplementary file 6 — Supplementary Material 6: Information on the implementation process of the 17 analysed documents [file 12913_2025_13563_MOESM6_ESM.docx]

Additional file 5 - Information on the implementation process of the 17 analysed documents

| **Document** | **Barriers** | **Facilitators** |
| --- | --- | --- |
| Patient safety strategy 2.0 | The strategy is based on a macro level, rather than on a micro level for a more detailed approach | Annual reports are published on patient safety at a federal level |
| Quality strategy for the Austrian healthcare system Version 2.1 | There is lack of human resources and not enough education and training is currently available to excel the quality of the strategy | The cross-professional and cross-sector patient safety strategy currently implied can open doors for further development in the care system |
| The Client and Patient Safety Strategy and Implementation Plan 2022–2026 | Not defined | Wellbeing at work for healthcare workers and other professionals |
| National Patient Safety Programme | Not defined | Not defined |
| 1^st^ roadmap 2023-2025 “Improving patient safety and residents”. A continuation of the national patient safety program 2013-2017 | Not defined | Not defined |
| Patient Safety Strategy 2019-2024 | Not defined | Not defined |
| National Plan for Patient Safety 2021 - 2026 | Not defined | Not defined |
| National Strategy for Health Quality 2015-2020 | Shortage of resources | Administration commitment |
| Patient Safety Strategy | Not defined | The Serbian law on healthcare establishes continuous improvement as one of it's principles. |
| National Strategy for Patient Safety in Healthcare (2023 - 2031) | Lack of human resources | Support and awareness of the urgency of its implementation by its stakeholders. |
| Patient Safety Strategy for the National Health System 2015-2020 | Pandemic | Involvement of the different stakeholders |
| National Action Plan for Increased Patient Safety in Swedish Health Care 2020-2024: Act for safer healthcare | Not defined | Stakeholders need to work together – municipally, regionally and nationally - collaboration and coordination. Follow-up in the field of patient safety - The follow-up must help to create motivation to continue to develop a high level of patient safety in all areas of healthcare. National measures are needed to support the implementation. In order to create strong implementation that contributes to increased patient safety, the following needs to exist at national level: a) access to experts who can contribute with knowledge and can provide education in patient safety; b) resources for research and development to both follow and develop knowledge in the field; c) resources to develop new tools and methods to both work with and to monitor safety in healthcare; d) a national platform and structure for the coordination and implementation of national measures and the feeding back of experiences. Take into account the ongoing patient safety work in the country, in order to prioritise actions and measures where the needs are greatest. At municipal and regional level, there needs to be: a) a safety proficient management that moves towards increased patient safety and at the same time creates the conditions for a good safety culture; b) sufficient staff with sufficient skills who understand and have knowledge of patient safety; c) safe and user-friendly technology; d) a sustainable working environment; e) ability to take advantage of the knowledge and experience of patients and relatives. In order to work towards the vision and the overall goal, four basic conditions are highlighted: a) Committed management and clear governance; b) A good safety culture; c) Adequate knowledge and competence; d) The patient as co-creator. |
| The NHS Patient Safey Strategy | Not defined | The existence of a dedicated, diverse and skilled workforce;  Support of regulation on patient safety across professional organisations; |
| National Safety and Quality Health Service Standards - 2nd edition | Not defined | Not defined |
| Improving safety and quality in health care - A strategic plan for action in WA 2024-2026 | Not defined | Not defined |
| The Canadian Quality and Patient Safety Framework for Health Services | Resources allocation; Human resources challenges; cultural competency; Technological barriers | Considered as a relevant topic and a priority in all care settings; Focus on patient and provider safety, regardless of the situation |
| Safer Together: A National Action Plan to Advance Patient Safety | Not defined | Not defined |
