# Supplementary material for: Understanding the development and implementation of national quality of care and patient safety strategic documents: a scoping review
Source: BMC Health Serv Res. 2025 Nov 27;25:1546. doi: 10.1186/s12913-025-13563-2 (PMC12681144; doi:10.1186/s12913-025-13563-2)
Supplement: Supplementary file 7 — Supplementary Material 7: Information on the evaluation/monitoring process mentioned in the 12 articles [file 12913_2025_13563_MOESM7_ESM.docx]

Additional file 6 - Information on the evaluation/monitoring process mentioned in the 12 articles

| **Article** | **Evaluation/Monitoring Process** |
| --- | --- |
| National patient safety consortium: learning from large-scale collaboration | - The Consortium had come far in a short time; thus, evaluating the initiative was critical. An evaluation action team developed a logic model and evaluation plan.  - Given the complexity of the structure, process and ambitious intended outcome, as well as the ebb and flow of participant involvement sometimes changing over the four years, evaluating the Consortium and the action plan would be a complex undertaking. The work had been iterative, organic and evolving, so the evaluation similarly needed to be flexible and adaptive.  - One of the guiding principles was ongoing evaluation of the Integrated Patient Safety Action Plan. Volunteers from 18 Consortium participant organizations, including patient partners, came together as an evaluation action team to develop an extensive logic model and evaluation framework and engaged an independent consultant to implement the evaluation plan. It was easy to concur that using the collective impact framework for measurement was the right model for this evaluation and that securing an independent expert to provide objectivity was the right thing to do. The purpose was not just about measuring outputs or outcomes; it was equally important to evaluate the processes and the collaboration.  - Progress against expected targets for each of the action plans was regularly reviewed and shared, and leads groups were regularly meeting to review priorities and opportunities for greater alignment. The Consortium also made sure to celebrate its milestones and collective accomplishments. This spirit, as well as the guiding principles, perhaps helped provide the intrinsic impetus to maintain momentum and the commitment of the coalition members. Although reporting on the remarkable progress was important, the meaningful measurement of the process, outcome and impact was equally important to the Consortium.  - Of course, progress needed to be demonstrated against goals and the achievement of outputs and outcomes to funders, members and, most importantly, patients and their families. However, it was about more than that; it was about evaluating and making sense of that progress.The evaluation plan included four key questions: How do we collaborate? This evaluation domain will investigate the Consortium's capacity for and approach to effective collaboration. This domain uses the collective impact lens to examine how participating organizations interact and collaborate and how these collaborations enable the actions set forth by the action plan. What has been done? This evaluation domain will investigate the execution of the actions set forth by the action plan and to what extent the outputs of those actions are used by players in the healthcare community. How well is it working? This evaluation domain will investigate the achievement of the goals set forth by the action plan and how meeting the goals facilitates an overall increase in system capacity to coordinate and improve patient safety. Is it making a difference? This evaluation domain will investigate the impact the Consortium has on patient safety through the implementation of the action plan over time.  - The evaluation included document review, key informant interviews, focus groups and an online survey. The external evaluators worked closely with the evaluation action team to coordinate evaluation activities, review and approve the evaluation methodology and tools and review and deepen early, interim and final findings and recommendations.  - At the conclusion of the action plan and Consortium in 2018, the findings and recommendations from the evaluation were shared and celebrated with the hundreds of individuals and organizations that participated in the four-year initiative. |
| A study of the implementation of patient safety policies in the NHS in England since 2000: what can we learn? | Not defined |
| The Better Care Plan: A blueprint for improving America’s healthcare system | Not defined |
| From accreditation to quality improvement-The Danish National Quality Programme. | Each goal is assessed by monitoring a panel of key quality indicators at national, regional, municipality and hospital level. Data are continuously presented on management dashboards. Indicator results are accessible for all stakeholders, including the public, to ensure transparency and enable benchmarking. |
| A Quality Strategy to Advance the Triple Aim in California’s Medicaid Program | Following publication of the inaugural QS in 2012, the QS team published its baseline QI inventory, which reflected QI activities underway in 2012. Follow-up inventories using similar survey and in-person interview methods as used to collect baseline data continued annually through 2018. The QS team documented 129 unique QI activities over 6 inventories from 2012 to 2018. The total number of activities was 44 at baseline, grew to 66 in 2013, and remained relatively stable until declining to 46 in 2018. The 2013 inventory documented the greatest number of new QI activities (n = 30), whereas the 2018 inventory documented the least new activities (n = 6). To identify organizational features that enhance or impede QI and measure possible changes in DHCS culture, the QS team administered an organizational QI maturity survey in January 2014 and 2015. The survey consisted of a 10-question subset of the valid and reliable QI maturity survey developed to evaluate the Robert Wood Johnson Foundation Multistate Learning Collaborative. The survey was distributed to DHCS executive staff during staff meetings and emailed to managers and supervisors. The National Quality Strategy established indicators for each priority and tracked progress on those indicators over time, highlighting select QI activities that support each indicator. Conversely, the DHCS QS identified a portfolio of QI activities for each priority, with each QI activity linked to its own indicators. This arrangement was meant to reduce performance pressure for DHCS program administrators and create an open door for participation in the QS. However, the lack of priority indicators limited quantification of QI progress. |
| Understanding the factors influencing implementation of a new national patient safety policy in England: Lessons from 'learning from deaths'. | Not defined |
| Quality improvement lessons learned from National Implementation of the "Patient Safety Events in Community Care: Reporting, Investigation, and Improvement Guidebook". | Not defined |
| The Danish health care quality programme: Creating change through the use of quality improvement collaboratives. | Not defined |
| Reporting and use of the OECD Health Care Quality Indicators at national and regional level in 15 countries | From 2003, OECD launched "Health Care Quality Indicators" project. It aims to measure and compare the quality of health systems.  There are indicators for care for chronic conditions and acute exacerbation, prescribing, patient safety, mental disorders, cancer and patient experiences with ambulatory care.  Most OECD countries (15 out of 17) used one or more OECD indicators on reports, either national or regional, for quality on healthcare. The most reports they perform, the more developed the healthcare system is. Most countries focus on reporting indicators on cancer or chronic care, followed by patient safety indicators. The use of this indicators allows comparison between diferent countries, since everyone is using the same way of measuring. |
| Quality improvement and accountability in the Danish health care system | Quality assessments are performed by data collection, auditing and learning activities, implementation of improvement and follow-up activities. The data is shared with professionals, getting them involved on the process and aggregated results are openly disclosed to the public. |
| Effects of the Italian Law on Patient Safety and Health Professional Responsibilities Five Years after Its Approval by the Italian Parliament | 2 years after the application of the law, a survey was conducted to evaluate it's impact on healthcare workers. Overall, the results showed that the law had a positive impact on communicating adverse events, on checklist adoption and participation in educational activities on healthcare risk management. It was a reliable educational tool to enhance patient safety culture. A new survey was conducted, after 5 years, of the law promulgation, for Clinical Risk Managers and Directors of Regional Centers.  The survey aimed to know how were the law requirements implemented; if there was a coordinating role at regional level and what role the had on COVID-19 pandemic; how were CRM organized; what kind and how many team members the had at their disposal; what kind of activities they performed; what chalenges they face and what interaction they have with the regional center and other hospitals. |
| Analysing 'big picture' policy reform mechanisms: the Australian health service safety and quality accreditation scheme | Not defined |
