# Supplementary material for: Understanding the development and implementation of national quality of care and patient safety strategic documents: a scoping review
Source: BMC Health Serv Res. 2025 Nov 27;25:1546. doi: 10.1186/s12913-025-13563-2 (PMC12681144; doi:10.1186/s12913-025-13563-2)
Supplement: Supplementary file 8 — Supplementary Material 8: Information on the evaluation/monitoring process of the 17 analysed documents [file 12913_2025_13563_MOESM8_ESM.docx]

Additional file 7 - Information on the evaluation/monitoring process of the 17 analysed documents

| **Document** | **Evaluation moment** | **Evaluators** | **Indicators** |
| --- | --- | --- | --- |
| Patient safety strategy 2.0 | Not defined | Not defined | Not defined |
| Quality strategy for the Austrian healthcare system Version 2.1 | Not defined | Not defined | Key Milestones:  1.1. a) Update patient safety strategy and implementation measures; b) Determining key topics (e.g.: Further developing the error reporting and l earning system; Defining and e stablishing risk management, qualification frameworks for health professions on the subject of patient safety and medication safety; c) Implementation of the defined measures on the key topics; d) ongoing review and, if necessary, adaptation of the measures taken; e) Preparation of the Austrian report on patient safety 1.2 a) Definition and implementation of hygiene measures, measures for reduction of antimicrobial resistance and prevention of health system-associated infections (HAI) 2.1 a) Operation and further development of A-IQI; b) Setting and calculating indicators; c) Further development of the indicators (including definition of new indicators); d) Conducting peer reviews; e) Preparation and initiation of the decided improvement measures until 2023; f) Continuation and further development of the subsidiary quality registers and incorporating selected indicators 2.2 a) Implementation and evaluation of the pilot projects that have already been initiated by the end of 2017; b) Gradual rollout of quality measurement in private medical practices across the entire country; c) Adaptation and further development of the concept into the method Quality measurement in the entire outpatient area; d) Gradual rollout of quality measurement throughout the outpatient area; e) Setting up a continuous reporting system including publication of appropriate results; f) Evaluate and, if necessary, adjust previously defined indicators and the quality improvement process based on the results and experiences from pilot projects; g) Developing and defining further indicators for the continuous improvement and expansion of the indicator set for the private medical sector, in particular building on the uniform diagnosis and performance documentation that is still to be implemented; h) Designing suitable public relations work that follows the principle of transparency; i) Definition of incentive systems for participation in quality circles from 2017 2.3 a) Preparing and carrying out the cross-sector survey using the methodology developed in the first target management period and the results and insights gained from it; b) Adding or specifying questions based on the defined goals and measures, e.g. on the topic of integrated care and chronic illnesses; c) Analysis and preparation of results including derivation of findings and measures; d) Publication of selected results in an appropriate form; e) Preparation and participation in ATHIS 3.1 a) Supporting healthcare providers/facilities in the implementation of the minimum requirements for quality management; b) step-by-step evaluation of the implementation of the minimum requirements quality management at selected health service providers 3.2 a) Continuation of the work begun on competency profiles (PHC); b) Implementing the competency profiles in the curricula for the priority areas; c) Develop and implement qualifications frameworks to provide guidance for all healthcare professionals 4.1 a) The updated nationwide quality standards for admission and discharge management as well as for preoperative diagnostics for elective; b) The method for developing federal quality standards in accordance with the Health Quality Act (including consideration of the interface to the social sector) has been revised; c) Completion, publication and target group-oriented dissemination of the quality standards that have already been started (diabetes mellitus, stroke, chronic pain, preventive colonoscopy, use of antibiotics); d) Periodic prioritization of additional topics for nationwide quality standards; e) Further nationwide quality standards for newly prioritized subject areas are being developed.; f) Implementation of quality standards; g) Evaluation and, if necessary, updating of quality standards 4.2 a) Identifying and prioritizing illnesses/needs for others IV measures; b) Implementation planning (based on framework specifications); c) Developing and implementing measures to promote IV; d) Evaluation, evidence based and quality assurance of IV measures 5. a) Continuation of work based on the evaluation of the “HTA strategy” (Deriving recommendations for action); b) gradually developing a strategic approach to the topic of evidence (EbM, HTA, EbPH) for Austria 6.1 a) Revising the questionnaires on quality systems in acute hospitals and inpatient rehabilitation facilities as well as developing a new questionnaire for independent outpatient clinics; b) Defining a standardized process for data exchange between www.kliniksuche.at and www.qualitiesplattform.at; c) Collection and evaluation of quality systems in independent outpatient clinics, acute hospitals and inpatient rehabilitation facilities; d) Involving other health service providers in the preparation of the next cross-sector quality reporting; e) Regular data c ollection via www.qualitiesplattform.at for kliniksuche.at; f) Preparation of the c oordinated periodic survey o n q uality systems i n healthcare facilities and publication of the results 6.2 a) Updating and maintaining information platforms; b) Further developing and expanding the information offering; c) Coordinating the content of the platforms |
| The Client and Patient Safety Strategy and Implementation Plan 2022–2026 | Not defined | Not defined | Ten indicators were selected for monitoring progress towards the goal of the Client and Patient Safety Strategy. These indicators of success provide information on the progress made in the strategic priorities and objectives in Finland. They also enable international benchmarking of the status of implementation of the WHO Global Patient Safety Action Plan in Finland. Monitoring the key indicators will commit Finland to the objectives specified by the WHO. By achieving these objectives, we can demonstrate that Finland has reached its goal of becoming a model for client and patient safety by 2026. Indicators: Indicator 1 (3.3) Service organisers are committed to implementing the objectives of the national Client and Patient Safety Strategy in their own strategies and action plans. Target level: By 2024, at least 60% of service organisers have recorded client and patient safety as one of their strategic objectives (or the inhabitants of committed wellbeing services counties account for 80% of the population). Source of data: Report by the Finnish Centre for Client and Patient Safety Indicator 2 (3.1) Service providers have adopted hotline indicators of Never Events as part of their monitoring system. Target level: In 2024, at least 60% of service providers are monitoring hotline reports of Never Events in real time. In 2026, at least 80% of service providers are monitoring hotline reports of Never Events in real time. Source of data: Report by the Finnish Centre for Client and Patient Safety Indicator 3 (4.3) The incidence of healthcare-associated infections (HCAIs) shows a downward trend. Target level: A reliable and comprehensive monitoring and reporting system for healthcare-associated infections is adopted by 2024. In 2026, the incidence of healthcare-associated infections shows a significant decrease. Source of data: Register data collected by the Finnish Institute for Health and Welfare Indicator 4 (4.1) The number of medication-related harm shows a downward trend. Target level: By 2026, a national indicator has been defined for medication safety and a monitoring system has been launched for this purpose. Monitoring data from 2029 shows a decline in the number of medication-related harmful events. Source of data: Finnish Medicines Agency Fimea and/or other national monitoring Indicator 5 (1.1 and 3.3) Cooperation models have been created between client and patient representatives and service providers and service unit leaders. Target level: By 2024, 30% of service providers have described and launched a cooperation model. By 2025, 60% of service providers have described and launched a cooperation model. Source of data: Report by the Finnish Centre for Client and Patient Safety Indicator 6 (2.1) The contents of the WHO Patient Safety Curriculum are included in the basic training of all healthcare and social welfare professionals. Target level: All medical and 80% of other curricula starting in 2024 correspond to the contents of the WHO Curriculum. Incorporated across all curricula in 2026. Source of data: Survey conducted by centres of expertise for university faculties of medicine, universities of applied sciences and other educational institutions. Indicator 7 (2.2) National development work has been launched to increase safety and wellbeing at work among healthcare and social welfare staff. Target level: All service organisers are committed to promoting the specified objectives by the end of 2024. The results will be evaluated in 2026. Source of data: Report by the Finnish Institute of Occupational Health Indicator 8 (1.2 and 3.3) The reporting and learning procedure for safety incidents has been reformed to meet the needs of a changing service system in terms of content and it has been integrated as part of service organisers’ information systems. Target level: A substantive and technical reform of the reporting system completed by 2025. 60% of service organisers have put the revamped system in place during 2026. Source of data: Report by the Finnish Centre for Client and Patient Safety Indicator 9 (3.1) Monitoring reports on client and patient safety are published annually at the national level and in the wellbeing services counties. Target level: National reports are published by the Finnish Centre for Client and Patient Safety and 60% of service organisers publish their own reports from 2024 onwards. By 2026, 80% of service organisers publish their own reports. Source of data: Report by the Finnish Centre for Client and Patient Safety. Indicator 10 (collaboration and networks) Networks promoting client and patient safety cover all stakeholders and geographically the whole country. Target level: Target for coverage achieved by the end of 2024. Source of data: Report by the Finnish Centre for Client and Patient Safety |
| National Patient Safety Programme | Not defined | Not defined | Not defined |
| 1^st^ roadmap 2023-2025 “Improving patient safety and residents”. A continuation of the national patient safety program 2013-2017 | Not defined | Not defined | Not defined |
| Patient Safety Strategy 2019-2024 | A National Patient Safety Programme has been established, which will oversee and monitor the implementation of this Strategy. | Not defined | Not defined |
| National Plan for Patient Safety 2021 - 2026 | Each year - online form; Evaluation moment at the middle of the application period. Finally, the final evaluation up to six months after the application period of the plan. | A designated commission, selected from stakeholders involved | 1. nº of institutions with an education program on patient safety;  2. nº of institutions with a safety culture evaluation implemented;  3. nº of institutions with, at least, one patient safety campaing for patients, families and carers;  4. Implementation of a national patient safety campaign;  5. nº of hospitals and primary care settings with patient safety indicators defined;  6. Publication of the legal framework update for the Quality and Safety Commissions;  7. nº of primary healthcare facilities with access to discharge reports online;  8. nº of units with electronic clinical information available (discharge and transference);  9. nº of institutions performing internal audits on communication in healthcare transitions; 10. Publication of the "Patient Consent for treatment" normative;  11. nº of institutions evaluating patients'perceptions on the "patient consent for treatment" form;  12. nº of public institutions with adverse events reports included on it's contracted targets; 13. Publication of the legal framework for the notifier protection; 14. nº of institutions reporting adverse events, using a report platform; 15. Development of a new version of the notification platform; 16. Performance on the development of the audit mode for the electronic platform, as well as the feedback to professionals and citizens. 17. nº of institutions using digital tools for safe practices on safe surgery, birth safety, falls, pressure ulcers, patient identification; medication safety and medication reconciliation; 18. nº of institutions using tools to monitor the risk for safety events on all levels of care, including homecare; 19. nº of institutions with contingency plans for public health emergencies; 20. nº of institutions with defined strategies to implement safe practices on safe surgery, birth safety, falls, pressure ulcers, patient identification; medication safety and medication reconciliation; 21. nº of institutions with annual internal audits performed and published; 22. nº of hospitals with epidemiological surveillance on healthcare associated infections, antimicrobial resistance and consumption; 23. nº of hospitals with an antibiotic stewardship program; 24. nº of new infections associated with the use of invasive medical devices vs. The number of days the device was used; 25. nº of cabapnem resistant K. Pneumonae identified vs. nº of non resistant K. Pneumonae in the same period; 26. nº of antibiotics used at the end of the 2026 vs. nº antibiotics used in 2021; 27. nº of hand hygene observations performed correctly (before contact with the patient) vs. nº of hand hygene observations. 28. Telehealth normative publication. |
| National Strategy for Health Quality 2015-2020 | Annually | Health directorate | Not defined |
| Patient Safety Strategy | Not defined | AZUS | Not defined |
| National Strategy for Patient Safety in Healthcare (2023 - 2031) | Regular, but not clearly definedt | Authors of the strategy/ Ministry of health | Indicators not selected, but there is a plan to select them: "The selection of indicators must be based on the country’s priority tasks, healthcare needs and existing data capacities". Indicators promoted by authors: a) indicators of dimensions of high-quality medical treatment; b) indicators of structure, processes and outcomes; c) indicators of diseases in individuals and the population. |
| Patient Safety Strategy for the National Health System 2015-2020 | For the evaluation of this strategy, indicators were defined after the document publication. They were reported in 2022 - *Manual para la evaluación de la estrategia de seguridad de paciente del sistema nacional de salud 2015-2020*. Evaluation at the end of the application period. | Subdirección General Calidad Asistencial. Dirección General de Salud Pública. Ministerio de Sanidad | Objective 1: Primary healthcare centers with a patient safety plan; Hospitals with a patient safety plan. Objective 2.1: Hospitals with high-risk medication protocols; Primary healthcare centers with a high risk medication protocol for critical patients; Hospitals with actions to promote medication safety; Hospitals that perform medication conciliation at discharge; Prescription patterns; Adverse events related with medication errors; number of actions on the Institute for Safe Medication Practices. Objective 2.2: Antiseptic solution for hand hygiene existence and consumption; Hand hygiene performance; Healthcare associated infections surveillance data. Objective 2.3: Safe surgery program adherence by hospitals; Professionals responding to the safety culture survey; Professionals with patient safety education; Performance on the surgical safety checklist; practices adjusted after the use of the surgical safety checklist; prophylactic thromboembolic medication adjust; post-surgery pulmonary thromboembolism; medication identification system used; readmissions after surgery; post-surgical venous thrombotic events; post-surgical deaths; hospitals with a doctor and a nurse responsible for the zero surgical infection program; global compliance on the zero surgical infection program; number of iatrogenic pneumothorax; number of post-surgical sepsis events; surgical wound dehiscence; post-surgical bleeding/bruise; retained surgical devices. Objective 2.4: Patient admitted with care plan; pressure ulcers; falls rate on hospitals; hospitals with a safe restraint protocol for patients. Objective 2.5: Hospitals with a correct patient protocol; Hospitals with a tracing and identification of samples protocol. Objective 2.7: Hospitals and primary care settings with a severe adverse events protocol. Objective 2.8: Paediatric units with education on ionizing treatments; Hospitals with a risk map for nuclear medicine and radiology units; Hospitals with an annual report on adverse events on radiology and nuclear medicine. Objective 3.1: Hospitals and primary care facilities with risk management units. Objective 3.2: Hospitals and primary healthcare facilities with an adverse event reporting system; number and ratio of adverse events reported; Reported adverse events with harm; Reported adverse events with no harm; Medication and ionizing radiation adverse events; Adverse events severity; anonymous adverse events reports; management of adverse events reports. |
| National Action Plan for Increased Patient Safety in Swedish Health Care 2020-2024: Act for safer healthcare | The evaluation process will include regular reporting through annual progress reports, monitoring using specific indicators, and conducting in-depth analyses in priority areas | The evaluators involve national health authorities, caregivers, and external stakeholders | Not defined |
| The NHS Patient Safey Strategy | Not defined | NHS England and NHS Improvement | Not specified/according to activities |
| National Safety and Quality Health Service Standards - 2nd edition | Not defined | Not defined | Not defined |
| Improving safety and quality in health care - A strategic plan for action in WA 2024-2026 | Not defined | Not defined | Not defined |
| The Canadian Quality and Patient Safety Framework for Health Services | Not defined, but recommendation is made for the evaluation to be made regularly. | Institutions can evaluate their own improvement on the program | Goal 1: 1.1. Patients’ ratings of the extent to which care was provided with respect; 1.2. Patient and provider involvement in care planning, governance, and evaluation; 1.3. : Patients’ overall ratings of health service experiences & Providers’ overall rating of health service experiences. Goal 2: 2.1. Assessment of organizational efforts to monitor, review, and address patient safety incidents & Training on quality improvement and patient safety provided at all organizational levels; 2.2. Rate of avoidable deaths (in and outside the hospital setting) & Rate of patient harm events; 2.3. Measures of safety culture, patient harm events and avoidable hospital readmissions are reported publicly. Goal 3: 3.1. Wait times for locally selected care, treatments, and procedures, analyzed by sociodemographic variables, geographic variables, and/or deprivation indices & Rate of access to primary care provider, analyzed by sociodemographic variables, geographic variables, and/or deprivation indices; 3.2. Provider skill mix, given health service guidelines and needs of the population served. Goal 4: 4.1. Health services based on needs assessment; 4.2. Variations in appropriate care, for locally selected interventions, are documented and inform quality improvement processes; 4.3. Implementation of health service innovation includes risk management, training, and evaluation to meet patient needs. Goal 5: 5.1. Communication between primary care providers and specialists; 5.2. Prevalence of electronic communication with patients & Prevalence of integrated electronic health records. |
| Safer Together: A National Action Plan to Advance Patient Safety | A separate document was elaborated regarding the evaluation process: Self-assessment tool - National Steering Committee for Patient Safety. Self-Assessment Tool: A National Action Plan to Advance Patient Safety. Boston, Massachusetts: Institute for Healthcare Improvement; 2020. | Not defined | Not defined |
